# Molecular Mechanisms of Bacterial Resistance to Antimicrobial Peptides in the Modern Era: An Updated Review

**DOI:** 10.3390/microorganisms12071259

**Published:** 2024-06-21

**Authors:** Layla Tajer, Jean-Christophe Paillart, Hanna Dib, Jean-Marc Sabatier, Ziad Fajloun, Ziad Abi Khattar

**Affiliations:** 1 Laboratory of Applied Biotechnology (LBA3B), Azm Center for Research in Biotechnology and Its Applications, Department of Cell Culture, EDST, Lebanese University, Tripoli 1300, Lebanon; layla1tajer@gmail.com (L.T.); ziad.fajloun@ul.edu.lb (Z.F.); 2 CNRS, Architecture et Réactivité de l’ARN, UPR 9002, Université de Strasbourg, 2 Allée Konrad Roentgen, F-67000 Strasbourg, France; jc.paillart@ibmc-cnrs.unistra.fr; 3 College of Engineering and Technology, American University of the Middle East, Egaila 54200, Kuwait; hanna.dib@aum.edu.kw; 4 CNRS, INP, Inst Neurophysiopathol, Aix-Marseille Université, 13385 Marseille, France; 5 Department of Biology, Faculty of Sciences 3, Lebanese University, Campus Michel Slayman Ras Maska, Tripoli 1352, Lebanon; 6 Faculty of Medicine and Medical Sciences, University of Balamand, Kalhat, P.O. Box 100, Tripoli, Lebanon

**Keywords:** antimicrobial resistance, antimicrobial peptides, molecular resistance, bacterial membranes, lipopolysaccharides, efflux pumps, mutations, peptide modifications, cationic peptides, host–pathogen interactions

## Abstract

Antimicrobial resistance (AMR) poses a serious global health concern, resulting in a significant number of deaths annually due to infections that are resistant to treatment. Amidst this crisis, antimicrobial peptides (AMPs) have emerged as promising alternatives to conventional antibiotics (ATBs). These cationic peptides, naturally produced by all kingdoms of life, play a crucial role in the innate immune system of multicellular organisms and in bacterial interspecies competition by exhibiting broad-spectrum activity against bacteria, fungi, viruses, and parasites. AMPs target bacterial pathogens through multiple mechanisms, most importantly by disrupting their membranes, leading to cell lysis. However, bacterial resistance to host AMPs has emerged due to a slow co-evolutionary process between microorganisms and their hosts. Alarmingly, the development of resistance to last-resort AMPs in the treatment of MDR infections, such as colistin, is attributed to the misuse of this peptide and the high rate of horizontal genetic transfer of the corresponding resistance genes. AMP-resistant bacteria employ diverse mechanisms, including but not limited to proteolytic degradation, extracellular trapping and inactivation, active efflux, as well as complex modifications in bacterial cell wall and membrane structures. This review comprehensively examines all constitutive and inducible molecular resistance mechanisms to AMPs supported by experimental evidence described to date in bacterial pathogens. We also explore the specificity of these mechanisms toward structurally diverse AMPs to broaden and enhance their potential in developing and applying them as therapeutics for MDR bacteria. Additionally, we provide insights into the significance of AMP resistance within the context of host–pathogen interactions.

## 1. Introduction

AMR claims the lives of around 700,000 people annually, solidifying its status as one of the most significant global health threats. Looking ahead to 2050, estimates suggest that deaths attributed to this issue could soar to 10 million annually, marking a staggering increase of roughly 14.3 times compared to the current levels [1]. AMR is also expected to raise the occurrence of diseases, amplify the seriousness of illnesses, escalate disability rates, and accordingly, become the world’s primary cause of death [2,3].

Many factors contribute to the spread of AMR, including the poor medication quality (such as counterfeit production), inappropriate prescription practices, patient misuse of antibiotics, inadequate surveillance systems and tests for AMR, and overuse of antibiotics in the industrial, animal, and agricultural sectors [4]. Wars and conflicts also play a crucial role in contributing to this grave situation [5]. Last, but not least, during the coronavirus disease 2019 pandemic, there was a surge in the incorrect overuse of antibiotics [6].

In light of this, AMPs have emerged as a solution to address this challenging situation [7], demonstrating their effectiveness in combating resistant microorganisms. AMPs primarily damage bacteria through a non-receptor-mediated membrane mode, enabling them to impede microbes’ ability to repair their damaged membranes [8] (for a detailed review on AMPs, see [9]). AMPs are ubiquitous across all life domains [10]. Currently, more than 3940 peptides have been documented in the AMP Database [11]. Upon administration, AMPs selectively target bacterial membranes, primarily due to the prevalence of anionic lipids, such as cardiolipin (CL) and phosphatidylglycerol (PG). Puzzlingly, the presence of CL has been observed to protect against AMP-mediated membrane disruption in some cases [12], whereas in other scenarios, membrane disruption appears to be intensified by CL [13]. The subsequent Section 3.5 will delve into further detail regarding this aspect.

The presence of zwitterionic lipids, like phosphatidylcholine, in mammalian cells reduces the affinity of cationic AMPs to their membranes [14]. However, AMPs can still interact with eukaryotic cell membranes to some extent and interact with intracellular organelles including mitochondria, which may have implications for their therapeutic efficacy and potential side effects [15,16]. CL is a major functional component of the inner mitochondrial membrane in eukaryotes [17]. Despite some similarities in composition and electric charge between the cell membrane and outer mitochondrial membrane, it remains uncertain if AMPs interact with the outer mitochondrial membrane in a manner similar to that with the cell or bacterial membrane. Nonetheless, it is known that, once inside cells, AMPs alter the permeability of both the outer and inner mitochondrial membranes, thus triggering the mitochondrial pathway. Consequently, AMPs that induce mitochondrial apoptosis often simultaneously modulate the permeability of both mitochondrial membranes in a synergistic manner [18].

**Figure 1:**
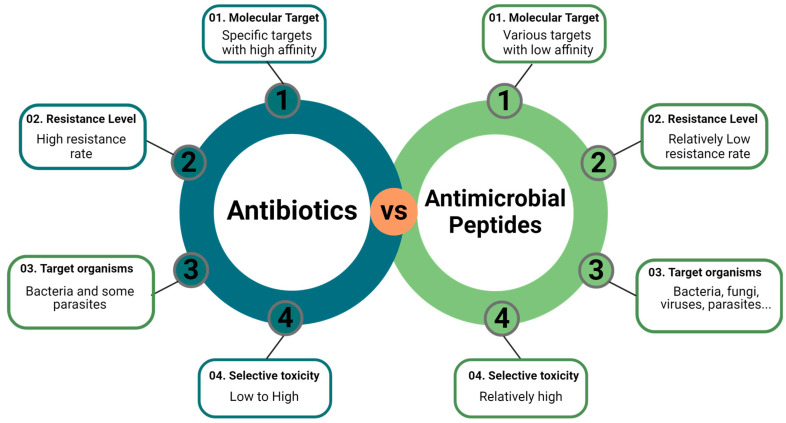
Advantages of AMPs over traditional antibiotics. This figure illustrates the differences between ATBs and AMPs regarding their target, resistance mechanisms, spectrum of activity, and cytotoxicity.

AMPs offer several advantages over traditional ATBs [11,14,19,20,21,22,23] (Figure 1), primarily due to their capability to target the challenging non-permeable double membrane of Gram-negative bacteria [24].

Bacteria have developed resistance to AMPs [25], although this occurrence is believed to be currently less widespread and progresses at a slower pace compared to what is observed with traditional antibiotics, apart from the non-host peptides polymyxins [22,26,27]. Indeed, Peschel and Sahl (2006) proposed that immune defense strategies using cationic AMPs as weapons and bacterial resistance mechanisms have co-evolved over millions of years. This co-evolution toward specificity and complexity enhances the fitness of bacteria [22]. However, hurdles in evolutionary adaptation to resist AMPs stem from various factors, such as functional compatibility, fitness costs, and the relatively lower occurrence of AMP resistance genes associated with mobile genetic elements [28]. Despite being in the gut microbiome, AMP resistance genes exhibit a diminished capacity for horizontal transfer comparing to antibiotic resistance genes [29]. In terms of fitness tradeoffs, studies have revealed that the increased expression of *mcr-1* gene, a plasmid-borne enzyme altering lipid A to confer acquired colistin resistance, results in many cellular alterations and reduced cell viability and growth rate [30]. The molecular mechanisms underlying acquired resistance through gene mutation(s) have been elucidated experimentally in several bacterial pathogens by isolating AMP-resistant strains following serial passages in the presence of gradually increasing AMP concentrations, or by directly exposing them to supra-minimum inhibitory concentration (MICs) of clinically relevant AMPs through plating (for a detailed review, see [31]). Therefore, the long-standing belief that acquiring resistance to AMPs is extremely difficult, and not a major concern [32,33,34,35] is mistaken, as such resistance can indeed develop at high rates at least under in vitro conditions. This results in mutants that do not entail significant loss of fitness [31], with sometimes significant levels of resistance granting cross-resistance to human AMPs inherent to innate immunity or those in clinical use, each with different structures and modes of action. Resistance to AMPs can manifest as constitutive (passive and intrinsic), with mechanisms rooted in the inherent properties of an organism that confer resistance even in the absence of AMPs. These properties, such as the components of bacterial membranes and cell walls, can be essential for the bacterial survival. Some bacteria like the Gram-negative species *Neisseria* spp., *Proteus* spp., *Providencia* spp., *Morganella morganii*, *Serratia* spp., *Edwardsiella tarda*, and *Burkholderia cepacia* complex (BCC) exhibit intrinsic resistance to polymyxins in vitro. This resistance is mainly due to the constitutive expression of lipopolysaccharides (LPSs) substituted with positively charged 4-amino-4-deoxy-L-arabinose (L-Ara4N), which electrostatically repulses polymyxins [36,37,38]. Such substitutions were shown to be essential for survial of *Burkholderia cenocepacia* since only conditional mutants in this pathway are viable. Furthermore, the members of BCC show exceptionally high resistance to polymyxin B and AMPs, with some isolates having MIC exceeding 512 μg/mL. This resistance is partly attributed to the synthesis of hopanoids (eukaryotic sterol analogs) and isoprenoids, which stabilize the inner membrane permeability, thus preventing the self-promoted uptake of polymyxin B. Active efflux pump expression and other mechanisms, not yet fully understood, also contribute to high polymyxin B resistance in *Burkholderia* [36,38,39]. 

On the other hand, pathogenic bacteria can modify their transcriptome in response to conditions encountered within their hosts [40], for an optimal adaptation to the presence of AMPs. Inducible (adaptive) resistance to AMPs is often governed by sophisticated systems, such as two-component regulatory systems (TCSs) [41,42,43,44], which are widespread among both commensal and pathogenic bacteria [45]. For example, in *Salmonella*, inducible resistance to AMPs is governed by the PhoPQ TCS, activated directly by sublethal concentrations of AMPs or indirectly by the stress caused by these peptides [46], as well as other stressors potentially encountered inside phagosomes, such as low magnesium concentrations or an acidic pH [41]. Additionally, PhoPQ activates the adaptor *pmrD* protein, which in turn activates the PmrAB TCS. This system is also induced in the presence of high Fe^2+^ concentrations to mediate various LPS modifications associated with resistance to cationic AMPs, including polymyxins [37,41,47].

Significant advances in structural biochemistry and microbial genetics have shed light on the structures of several AMPs and the molecular and mechanistic bases of bacterial resistance to these peptides. For example, Groisman et al. (1992) demonstrated that a single gene, *phoP*, encoding a transcriptional regulator, could render the intracellular pathogen *Salmonella* Typhimurium resistant to AMPs by modifying its outer membrane [48].

In this review, we thoroughly investigate and discuss the molecular mechanisms experimentally proven to confer AMP resistance in bacteria, whether constitutively or induced by environmental signals including AMPs themselves. Furthermore, we analyze their correlation with the virulence phenotype and pathogenicity determinants observed through in vitro cell culture bioassays and in animal models of infection.

## 2. Antimicrobial Peptides

AMPs are typically small molecules comprising fewer than 100 amino acid residues [49] and play a vital role in innate immunity, serving as the first line of defense against invading microorganisms within various host organisms [50]. AMPs are synthesized in different locations depending on the organism producing them. For example, mammalian AMPs are synthetized in mucosal tissues, glandular cells, the skin and colostrum, endothelial cells, and certain immune cells, such as neutrophils, macrophages, and dendritic cells [51].

**Figure 2:**
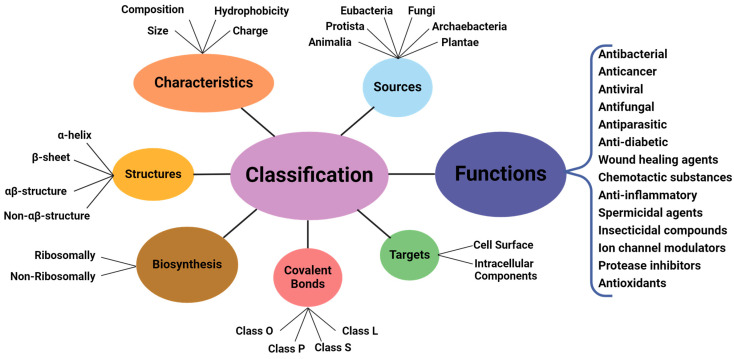
Classification of antimicrobial peptides. AMPs are classified according to seven criteria. The classification of AMPs based on their three-dimensional structure encompasses four categories: alpha (α), beta (β), alpha–beta (α-β), and non-α-β. These categories are defined by the presence of helical structures, β strands, a combination of helical and β structures, or the absence of both α and β structures, respectively. Regarding covalent bond classification, peptides are divided into four groups based on their polypeptide chain-bonding patterns: Class O peptides adopt circular structures due to a bond between the N-terminal and C-terminal backbone atoms; class P peptides take on a shape resembling the letter “P”, formed by a bond between the side chain of one amino acid and the backbone of another; class S peptides exhibit bonds between different side chains; and class L includes all linear peptides [11].

AMPs can be classified according to seven criteria, including targets, sources, characteristics, biosynthesis, structures, covalent bonding forms (universal classification), and ultimately, their predominantly antibacterial functions [11,52] (Figure 2).

Roughly 88% of AMPs are positively charged and display hydrophobic characteristics [11]. Consequently, AMPs interact with the negatively charged components of bacterial membranes, thereby disrupting them and ultimately killing microbes [53]. These AMPs have the capability to target multiple sites on both the membrane and within the bacterial cell [54,55]. Eradicating bacteria by AMPs can be achieved directly or indirectly. In the direct mode, AMPs target the membrane, either by binding to cytoplasmic membrane receptors [56] or by acting without direct binding [57]. In the latter case, the membrane is disrupted non-specifically, leading to damage through pore formation [58] or the permeation of cytoplasmic membranes without pore formation [59]. The pore formation mode is further categorized into two forms: the Barrel–Stave pore model and the toroidal pore model, while the non-pore formation mode is exemplified by the carpet model [60]. Additionally, AMPs can directly target the cell walls [58] or the intracellular components (DNA, RNA, and proteins) of bacteria [61]. Alternatively, the indirect mode involves modulating the immune system [62]. 

While the advantages of AMPs surpass those of classical ATBs, they also pose challenges, such as restricted bioactivity, potential biotoxicity, delivery, and the risk of hemolysis, particularly due to imprecise damage and failure to distinguish between foreign bodies and host cells [63,64]. For instance, defensins and cathelicidins are the two prominent mammalian families of cationic AMPs with amphipathic properties. Defensins are characterized by their cysteine-rich nature and the formation of β-sheet structures held together by disulfide bonds [65]. On the other hand, cathelicidins lack disulfide bonds and instead adopt amphipathic α-helical structures. In humans and mice, only one cathelicidin member exists, known as LL-37 and murine cathelicidin-related antimicrobial peptide (mCRAMP), respectively. The positive charge of these AMPs facilitates their selective interaction with negatively charged bacterial membranes [65,66]. LL-37 and defensins are produced in the bone marrow and epithelial cells [67] and have been observed to interact with host cell receptors even at concentrations significantly lower than the MIC of these peptides. This interaction contributes to various illnesses, including pulmonary disorders, autoimmune diseases, tumors, and cardiovascular and neurodegenerative diseases [68].

Additionally, AMPs are costly, poorly stable, and have a short half-life due to environmental factors, such as protease activity (mainly pancreatic enzymes), temperature, salts, extreme pH conditions, and reduced permeation through gastrointestinal membrane, which makes their oral administration much difficult [69,70,71,72,73]. Consequently, more than 50% of identified AMPs undergo modifications depending on their administration route and delivery system [74,75]. In contrast to other administration routes, such as intramuscular or subcutaneous routes, the stability requirement for peptides may be less stringent. Therefore, injection stands out as the preferred route of administration for most AMPs [76]. However, intravenous administration exposes peptides to the esterase and peptidase activity found in serum [77,78]. Moreover, optimizing the drug dosage and minimizing systemic exposure can reduce systemic side effects when administering a drug via the lungs. Inhaled peptide medications have shown a superior efficacy in terms of rapid onset [79]. The most prevalent and well-developed applications of AMPs involve their incorporation into nanoparticles, hydrogels, creams, gels, and ointments for topical use [80]. Further research is necessary to explore new and suitable administration routes.

Natural peptides serve as a template for creating synthetic peptides with enhanced stability, increased bioactivity, reduced toxicity, and additional antimicrobial activities [81,82]. These synthetic peptides can be generated through various modifications, including chemical alterations, targeted peptide mutagenesis, nanoengineering techniques, capping, motif usage, coupling with photosensitizers, or combination with ATBs [83,84]. However, approaches to enhancement should be pursued with caution and precision, as even minor alterations can compromise effectiveness.

For instance, lipidation involves attaching fatty acids to the active regions of the peptides [85]. The lipid addition confers numerous advantages to AMPs without altering their fundamental characteristics, such as increasing hydrophobicity, enhancing membrane interaction, improving penetrability, reducing degradation (thus increasing stability), and preserving cationic charges [86,87,88]. Nevertheless, this mechanism may also have significant drawbacks. For example, the elongated fatty acid chain increases hydrophobicity, leading to a greater toxicity against mammalian cells [89]. Additionally, it promotes self-assembly in water, resulting in a reduced interaction between peptides and the membranes of their targets [90].

Notwithstanding their potent antimicrobial effects in vitro, only some AMPs derived from bacteria, including polymyxin B, polymyxin E (commonly known as colistin), and bacitracin, are currently used to treat Gram-negative infections in humans, while others have shown promise in preclinical studies and clinical trials [91,92,93] (Table 1). Despite concerns regarding toxicity and the emergence of resistance [94,95,96], colistin has been reintroduced for use as monotherapy or in combination as a last resort for treating MDR and extensively drug-resistant (XDR) Gram-negative bacteria isolated from nosocomial infections. These infections involve pathogens such as *P. aeruginosa*, *Acinetobacter baumannii*, *K. pneumoniae*, and *E. coli* [97,98]. Because of poor pharmacokinetics, the intravenous administration of colistin is typically restricted to treating urinary tract infections or employed as inhalation therapy [99,100]. Efforts to optimize its use, including dosage adjustment and combination therapy with other antibiotics, are ongoing to balance its effectiveness with the risk of adverse effects.

## 3. Mechanisms of Resistance to AMPs

**Figure 3:**
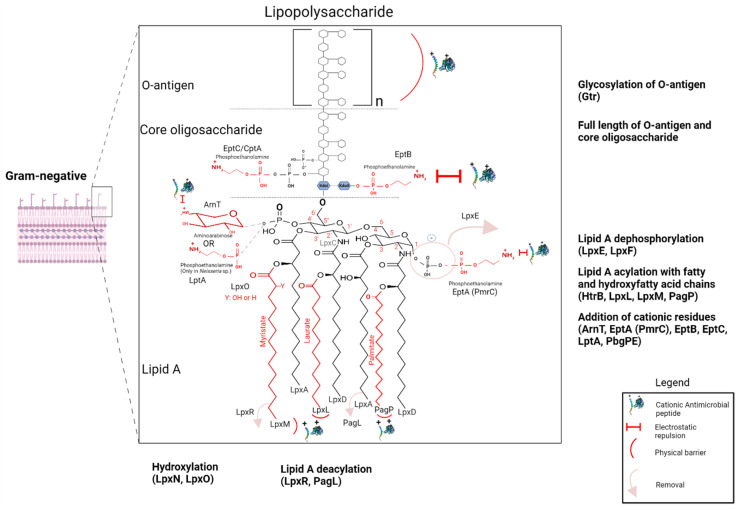
Modifications of LPS for enhanced resistance against AMPs. The position of a substituent or branch is identified by the number of the carbon atom it is bonded to.

**Figure 4:**
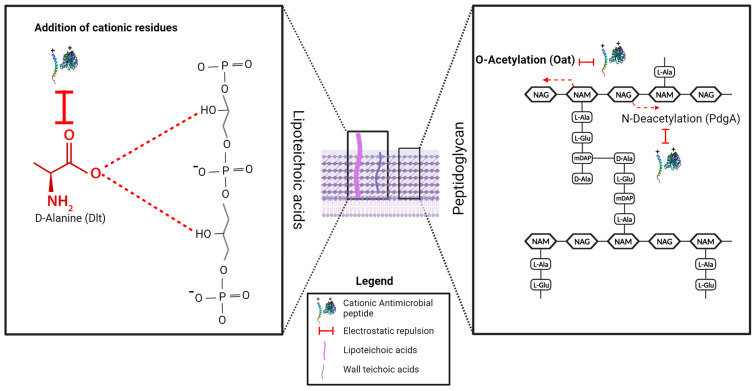
Modifications of teichoic acids, lipoteichoic acids, and peptidoglycans associated with AMP resistance. Enzymes involved in the modification reaction are mentioned between parentheses.

**Figure 5:**
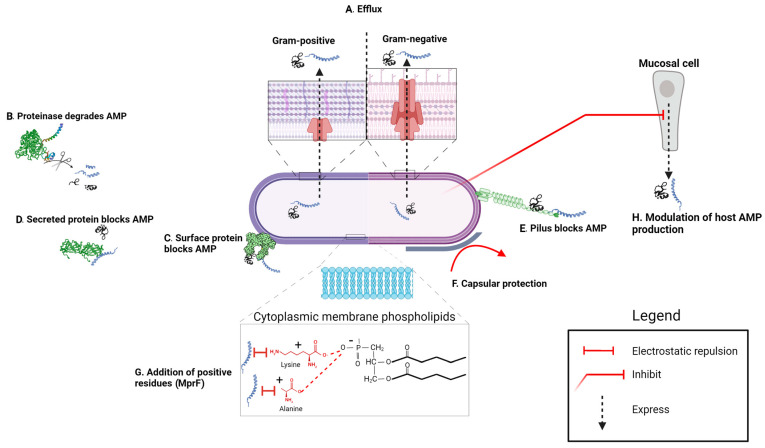
Bacterial mechanisms of AMP resistance not associated with modifications of cell wall structures. (**A**): Active efflux. (**B**): Secreted proteinases degrading AMPs. (**C**): Surface proteins trapping/inhibiting AMPs. (**D**): Secreted proteins blocking/inhibiting AMPs. (**E**): Pili-mediated blocking of AMPs. (**F**): Capsule-mediated protection. (**G**): Modifications of cytoplasmic membrane phospholipids by the incorporation of cationic residues. (**H**): Transcriptional downregulation of AMP production by mucosal host cells.

In this section, we explore the mechanisms of AMP resistance (Figure 3, Figure 4 and Figure 5), emphasizing their correlation with the bacterial morphological and functional structures encountered by AMPs from their exposure in the bacterial environment to their interaction with and traversal across bacterial envelopes until reaching the cytoplasmic membrane or other intracellular targets [66]. To reach their targets, AMPs must interact with and traverse the enveloping structures, which can vary in chemical and physical properties among bacteria. Understanding these interactions is crucial for elucidating the effectiveness of AMPs and devising strategies to overcome bacterial resistance.

Table 2 and Table 3 summarize the mechanisms of resistance to AMPs described for Gram-negative and Gram-positive bacteria and their association with virulence and pathogenicity.

### 3.1. Extracellular Proteolytic Degradation

Many bacterial pathogens employ surface-secreted or surface-expressed proteases to recognize and cleave cationic AMPs, allowing them to evade their bactericidal effects [126] (Figure 5B). These enzymes can be divided according to their active sites in five classes: cysteine proteases, aspartic proteases, metalloproteases, serine proteases, and threonine proteases [127]. The simple linear form of the α-helical peptide LL-37 and other cationic AMPs renders them susceptible in vitro to various proteases produced by different pathogenic bacteria (Table 2 and Table 3) (Figure 5B). In particular, *S.* Typhimurium produces an endopeptidase in the outer membrane, named PgtE, which specifically degrades the linear cationic AMPs LL-37 and C18-G. The transcription or outer membrane localization of PgtE is regulated by an unknown post-transcriptional mechanism activated by PhoPQ [128]. PgtE exhibits structural characteristics consistent with those of the OmpT or VII proteases of *E. coli* [129], and Pla from *Yersinia* [130]. The ability of *E. coli* to produce PgtE and degrade protamine is associated with the rapid uptake of K^+^ ions by bacteria [131]. However, the PgtE protease does not enhance *Salmonella* resistance to AMPs that adopt amphipathic β-sheet conformations stabilized by intramolecular disulfide bridges (e.g., defensins and protegrins), as these AMPs are resistant to protease activity.

**Table 2:** Mechanisms of AMP resistance in Gram-negative bacteria and their correlation with virulence and pathogenicity.

Bacteria	Mechanisms/Regulatory Pathways of Resistance	Gene(s) Involved	AMPs	Correlation with Pathogenicity/Virulence *	Refs.
*Acinetobacter* *baumannii*	Acylation of lipid A	*lpxL*	Polymyxin B	Unknown	[132]
	LPS-full length-mediated protection	*lpxA*, *lpxC*, and *lpxD*	Lysozyme, LL-37, and lactoferrin	Bacteremia (mice)	[133,134,135]
	Deacylation of lipid A	*naxD* (*pmrB)*	Polymyxin B	Unknown	[136]
	Hydroxylation of lipid A	*lpxO/pagQ*	Polymyxin B, colistin, and HBD-3	Bacteremia (*Galleria mellonella*) Antiphagocytic (invertebrates and mammalian cells)	[137]
	Addition of PEtN to lipid A	*ept, mcr* (plasmid-encoded), (*pmrAB* and *stkSR*)	Colistin	Increased virulence in *G. mellonella*	[138,139,140,141,142,143]
	Uptake by/binding to porins	*ompA* *ompW*	Colistin (uptake) LL-37, BMAP-28 (binding), and colistin	Virulence to the human airway epithelium, adherence to cells, and biofilm formation	[144,145]
	Active efflux (MFS and RND-types)	*emrB* *adeABC*	Colistin Colistin heteroresistance	Overexpression increased virulence in a pulmonary infection model	[146,147,148]
	Manipulation of host AMP production	*lpxO*	Galiomycin, gallerimycin, and lysozyme	Bacteraemia (*G. mellonella*)	[137]
*Actinobacillus* *pleuropneumoniae*	Outer membrane permeability	*ompW* (*soxS*)	Polymyxin B	Unknown	[149]
	Active efflux (ABC family/K^+^ dependent)	*sap*	PR-39	Respiratory tract infection (mice)	[150]
*Bordetella* sp.	O-antigen-mediated protection	*wlbA* and *wlbL* (*bvgAS*)	Cecropin, magainin, protamine, and melittin	Tracheal colonization (turkey)	[151,152]
	Acylation of lipid A	*pagP* (*bvgAS*) and *lpxL1*	C18G	Respiratory tract infection (mice) Infection of human macrophages	[153,154]
*Brucella abortus*	Dephosphorylation of lipid A	*lpxE*	Polymyxin B	Not required	[155]
*Brucella melitensis*	Active efflux (ABC-type)	*yejABEF*	Protamine, melittin, polymyxins, HBD-1, and HBD-2	Survival in macrophages (mice)	[156]
*Burkholderia* *cenocepacia* complex	Degradation by zinc metalloproteases	*zmpB* and *zmpA* (*cepIR* and *cciIR*)	HBD-1 and LL-37, respectively	Chronic respiratory infection (mice)	[157,158]
	Inhibition by exopolysaccahrides (mainly cepacian)	*-*	Cathelicidins	Lung infections of cystic fibrosis (humans)	[159]
	Blockage of AMP uptake by the LPS-heptosylated core oligosaccharide	*waaF*	Polymyxin B, melittin, and HNP-1	Unknown	[36,160]
	Stabilization of the inner membrane lipids	*ispH* (LytB; isoprenoid synthesis) and *hpnJ* (encodes hopanoid)	Polymyxin B	Unknown	[36]
	Protease-mediated protection (unknown role/mechanism)	*mucD* (HtrA protease family)	Polymyxin B	Unknown	[36]
	Active efflux (MATE-type)	*norM*	Polymyxin B	Unknown	[161]
	Addition of 4AraN to lipid A	*ugd_BCAL2946_* (two *ugd* in *B. caepacia*)	Polymyxin B	Unknown (mutants of 4AraN synthesis not viable)	[160]
	Alternative sigma factor regulon (37 °C)	*rpoE*	Polymyxin B	Phagolysosomal fusion in macrophages	[36]
*Campylobacter jejuni*	LOS-heptosylated core-mediated protection	*waaF*	Polymyxin B, HNPs, LL-37, and BPI	Invasion of INT407 cells in vitro	[162,163]
	LOS core-mediated protection	*galU*	Polymyxin B, colistin, magainin, cecropin, and bacitracin	Unknown	[164]
	Active efflux (RND-type)	*cme*	Polymyxin B	Intracellular survival and multiplication (*Acanthamoeba polyphaga)*	[165,166]
*Capnocytophaga* *canimorsus*	Dephosphorylation of lipid A	*lpxE*	Polymyxin B	Unknown	[167]
*Enterobacter cloacae* complex	Active efflux (RND-type)	*acrAB-tolC* (*soxSR*) *kexD/*(*crrC*)	Polymyxin B and colistin	Systemic infection (intraperitoneal mouse model)	[168,169,170]
	Addition of 4AraN to lipid A	*arn* (*phoPQ* and *mgrB*)	Colistin heteroresistance	Unknown	[171,172]
	Addition of PEtN to lipid A	*mcr*	Colistin	Unknown	[173]
	Potential efflux mechanism mediated by an inner membrane protein	*dedA (ecl)*	Colistin heteroresistance	Unknown	[169]
*Erwinia chrysanthemi*	Active efflux (ABC family/K^+^-dependent)	*sap*	α-thionin and anakin	Unknown	[174]
*Escherichia coli*	Protease-mediated degradation	*degP*	Lactoferrin	Urovirulence (humans)	[175]
		*ompT* and *degP*	Protamine, C18G, and LL-37		[131,176,177,178]
	Core oligosaccharide-mediated protection	*pmrD*	Polymyxin B	Unknown	[179]
	Acylation of lipid A	*pagP* and *lpxM* (*pmrAB* and *mgrB*)	LL-37	Unknown	[180,181]
	Addition of 4AraN to lipid A	*arn* (*phoPQ*, *pmrAB*, and *mgrB*)	Polymyxin B and colistin	Unknown	[182,183,184]
	Addition of PEtN to lipid A	*eptA*, *eptB*, *eptC*, and *mcr* (*phoPQ*, *pmrAB,* and *mgrB*)	Polymyxin B and colistin	Unknown	[184,185,186]
	Decreased entry via porins	*ompF*	Colicin and P6	Unknown	[187,188]
	Peptidoglycan modification	*amiA* and *amiC* (*cpxRA* and *nlpE*)	Protamine, magainin, and melittin	Unknown	[189]
	Active efflux (ABC-type)	*macB*	Bacitracin and colistin	Unknown	[190]
	Active efflux (RND and MFS-types)	*acrAB* and *emrAB* (*cpxRA*)	Protamine	Unknown	[191]
	Transcriptional repression of host’s AMP production (ETEC)	*elt* (heat-labile toxin-encoding gene)	HBD-1 and LL-37	Downregulation of kinase A, ERK MAP Kinase, and Cox-2 pathways (intestinal epithelial cells)	[192]
*Francisella novicida*	Dephosphorylation of lipid A	*lpxF*	Polymyxin B	Pulmonary and subcutaneous infections (mice)	[193,194]
*Francisella tularensis*	Deacylation of lipid A	*naxD*	Polymyxin B	Intracellular replication (mice)	[195]
*Haemophilus ducreyi*	Active efflux (ABC family/K^+^ dependent)	*sapA*	LL-37	Chancroid (human)	[196]
	Active efflux (RND-type)	*mtr*	LL-37 and β-defensins	Unknown	[197]
*Haemophilus influenzae*	Acylation of lipid A	*lpxL* (*htrB*)	Polymyxin B	Colonization of human airway epithelial cells	[198,199]
	Active efflux (ABC family/K^+^-dependent)	*sapA*	Canine β-defensin-1, HBD-1, -2, -3, LL-37, HNP-1, and melittin	Otitis media (chinchillas)	[200]
*Helicobacter pylori*	Dephosphorylation of lipid A	*lpxE* and *lpxF*	Polymyxin B, LL-37, HBD-2, and P-113	Gastrointestinal infection (mice)	[201]
	Addition of PEtN to lipid A	*eptA*	Polymyxin B	Unknown	[202]
	O and N-acetylations of peptidoglycan	* patA * and *pgdA* (synergistic)	Lysozyme	Stomach colonization (mice)	[203]
*Klebsiella pneumoniae*	Capsule-mediated protection	*cps*	Polymyxin B and lactoferrin	Pulmonary infection (mice)	[204]
	O-antigen-mediated protection	*wcaI*, *cpsB*, *wcaJ*, and *cpsG*	Histones	Unknown	[205]
	Acylation of lipid A	*lpxM* *lpxL2* and *pagP* *(mgrB* and *crrab*)	Polymyxin B, colistin, CP28, and C18G Polymyxins (*lpxL2*, *pagP*, *crrab*) HNP-1, HBD-1, -2, and -3 (*mgrB*)	Antiphagocytic, limits the activation of inflammatory responses by macrophages, and survival (*G. mellonella*); pneumonia (mice)	[143,206,207]
	Hydroxylation of lipid A	*lpxO* (*phoPQ*)	Polymyxins	Pulmonary infection (mice)	[208]
	Addition of 4AraN to lipid A	*pmrHFIJKLM* (*phoPQ*, *pmrAB*, and * mgrB*)	Colistin	Same phenotypes as for *lpxM* mutant in *G. mellonella*	[183,184,209,210]
	Addition of PEtN to lipid A	*eptA*, *eptB*, *eptC*, and *mcr*, (*phoPQ*, *pmrAB*, and *mgrB*)	Colistin	Unknown	[184,209,210,211]
	Activation of unknown systems dedicated to ameliorating AMP cytotoxicity	*ompA*	Polymyxin B and protamine	Pulmonary infection (murine)	[212]
	Active efflux (ABC family/K^+^-dependent)	*sapA*	LL-37	Systemic infection (mice)	[213]
	Active efflux (RND-type)	*acrRAB* *H239_3064* (*crrAB*)	Polymyxin B, HNP-1, HBD-1 and HBD-2, and colistin	Pneumonia (mice)	[214,215]
*Legionella pneumophila*	Acylation of lipid A	*rcp* *pagP* (*phoPQ*)	LL-37 Polymyxin B and C18G	Pulmonary colonization and infection (mice)	[216]
*Neisseria gonorrhoeae*	Inhibition by lactoferrin-binding protein B	*lbpB*	Lactoferricin	Unknown	[217,218]
	Inhibition by type IV pili adhesins (Same scenario in *N. meningitides*, involvement of host cell RhoA and Cdc42 signalling)	*pilE*	LL-37	Adherence to human epithelial cells	[219,220]
	Active efflux (RND-type)	*mtr*	LL-37, PG-1, PC-8, polymyxin B, and colistin	Genital tract infection (mice)	[221,222,223,224]
*Neisseria meningitidis*	Inhibition by lactoferrin-binding protein B	*lbpB* (*nalP*)	Lactoferricin	Unknown	[217,218,225]
	Sequestration/shielding by capsule	*cps*	HBD-1 and -2, HNP-1 and -2, LL-37, CRAMP, PG-1, and polymyxin B	Meningitis (humans)	[226,227]
	Sequestering by blebs from the OM and biofilm formation	*-*	Cationic AMPs	Unknown	[228]
	Addition of PEtN to lipid A (constitutive) (Same scenario in *N. gonorrhoeae*)	*eptA* (formerly *lptA*) (*misRS*) *dsbA*	Polymyxin B	Colonization, inflammation, and survival in neutrophils (EptA)	[229,230]
	Porin-mediated export	*porB*	Polymyxin B	Unknown	[229]
	Active efflux (RND-type)	*mtr* (constitutive)	Polymyxin B, PG, and LL-37	Unknown	[229]
*Photorhabdus laumondii*	Active efflux (RND-type)	*acrAB*	Polymyxin B and colistin	Slight effect on virulence (insects)	[231,232]
	Addition of 4AraN	*pbgPE* (*phoPQ*)	Polymyxin B, colistin, cecropins A and C	Septicemia and virulence (insects)	[233,234]
*Porphyromonas* *gingivalis*	Dephosphorylation of lipid A	*lpxF*	Polymyxin B	Unknown	[233,234,235]
	Outer membrane OmpA-like porins (undefined mechanism)	*pgm6* and *pgm7*	HBD-1 and -3, and LL-37	Unknown	[236]
	Inactivation by proteases RIA, RIB, and Kgp	*prpR1* and *kgp*	Cecropin B, brevinin, cecropin A 1-7, melittin 2-9, and mastoparan	Unknown	[237]
	Proteolytic degradation by gingipains (serine proteases)	*rgpAB*	Cecropin B	Abscess formation (mice)	
*Prevotella* sp.	Proteolytic degradation	Unknown	Cecropin B and brevinin	Unknown	[237]
*Proteus mirabilis*	Degradation by metalloprotease	*zapA*	LL-37	Urinary tract infection (mice)	[238]
	Addition of 4AraN to lipid A	*pmrAB*	Polymyxin B	Unknown	[239]
	Active efflux (ABC family/K^+^-dependent)	*sap*	Protegrin	Unknown	[239]
*Pseudomonas* *aeruginosa*	Degradation by elastase	*las*	LL-37	Corneal infection (mice)	[240]
	Capsule-mediated protection	*cps*	Polymyxin B	Resistance to neutrophil-mediated killing	[241]
	Shedding of host proteoglycans	*lasA*	LL-37 and human α-defensins	Pulmonary infection (mice)	[242,243]
	Hydroxylation of lipid A	*lpxO*	Polymyxin B	Acquisition of loss-of-function mutations during chronic CF lung infection (mice)	[244,245]
	Addition of 4AraN to lipid A	*pmrHFIJKLM* (*pmrAB*, *phoPQ*, *parRS*, *colRS*, and *cpsRS*)	Colistin and polymyxin B	Cystic fibrosis (humans)	[246,247,248]
	Addition of PEtN to lipid A	*eptA* (only by ectopic expression in L-Ara4N-defective mutants) and *mcr*	Colistin	Unknown	[249,250,251]
	Alteration of membrane phospholipid composition	*PA0920*	Protamine	Unknown	[252,253]
	Active efflux (RND family)	*mexAB, mexCD,* and *mexXY*	Colistin and polymyxin B	Controversial and opposing roles since some mutations increase virulence	[254,255,256]
	Stimulation of host cathepsins	Unknown	LL-37, HBD-1, and HBD-3	Cystic fibrosis (humans)	[257,258]
*Pseudomonas* *fluorescens*	Alteration of cytoplasmic membrane lipid composition	Unknown	Polymyxin B	Unknown	[259]
*Rhizobium etli*	Dephosphorylation of lipid A	*lpxE* and *lpxF*	Polymyxin B	Unknown	[167]
*Rhizobium* *leguminosarum*					
*Salmonella enterica*	Endopeptidase-mediated degradation	*pgtE* (*phoPQ*)	LL-37 and C18-G	Unknown	[128]
	Acylation of lipid A	*lpxM* (*msbB*) *pagP* (*phoPQ*)	Polymyxin B C18G and PG-1	Inflammation and septaecemia in mice (*msbB*), minor effects (*pagP*), Required for full virulence (*phoP*)	[182,260]
	Dephosphorylation of lipid A	*pagL* and *lpxR* (*phoPQ*)	Polymyxin B	Minor effects in mice (*pagL* and *lpxR*)	[261]
	Hydroxylation of lipid A	*lpxO* (*phoPQ*)	LL-37	Increased invasion of human epithelial cells and full virulence in animals (*lpxO* mutant)	[262]
	Addition of 4AraN to lipid A	*pmrHFIJKLM* (*phoPQ*)	Defensins and polymyxin B	Gastrointestinal infection (mice)	[260,263]
	Addition of PEtN to lipid A/core oligosaccharide	*eptA*, *eptBC* (core), *cptA* (core) (*pmrAB*), *mcr*	Polymyxin B and colistin	Unknown	[185,264]
	Peptidoglycan modification	*amiA* and *amiC* (*cpxRA* and *nlpE*)	Protamine, magainin, and melittin	Unknown	[189]
	Active efflux (ABC transporter)	*macAB*	C18G	Intracellular survival (macrophages)	[265,266]
	Active efflux (ABC family/K^+^-dependent)	*sap* *yejABEF*	Protamine, melittin, and polymyxin B	Gastrointestinal infection (mice)	[48,267,268]
	Extracytoplasmic σ^E^ factor	*rpoE*	P2, polymyxin B, and murine α-defensin cryptdin 4	Gastrointestinal infection (mice)	[269,270]
*Shigella dysenteriae*	Manipulation of host AMP production	Unknown	LL-37	Bacillary dysentery (human)	[271]
*Shigella flexneri*	O-antigen-mediated protection	Unknown	Histones	Unknown	[205,272]
	Alteration of host AMP production	*ospF* (*mxiE*)	Rabbit α-defensin NP5	Repression of NF-kB-responsive genes (Caco-2 and HeLa cells, rabbits)	[273]
*Ureaplasma parvum*	Host chromatin alterations	Unknown	HBD-1, HNP-6, and LL-37	Decreased histone H3K9 acetylation (Human THP-1 monocytoid tumor cell line)	[274]
*Vibrio cholerae*	Acylation of lipid A	*lpxL*	Polymyxin B	Unknown	[275]
	Hydroxylation of lipid A	*lpxN*	Polymyxin B	Unknown	[275]
	Sensing by OMP/Activation of a DegS-dependent σ^E^ factor	*ompU/degS/rpoE*	Polymyxin B and P2	Bile resistance in vivo (*ompU*)	[276,277]
	Active efflux (RND-type)	*vexAB*, *vexCD*, and *vexIJK*	Polymyxin B	Small intestine colonization (mice)	[278]
	Transcriptional repression of host’s AMP production	*ctxA* and *ctxB* (cholera toxin-encoding genes)	LL-37	Downregulation of kinase A, ERK MAP Kinase, and Cox-2 pathways (intestinal epithelial cells)	[192]
*Vibrio vulnificus*	K^+^ uptake transporter system	*trkA*	Protamine and polymyxin B	Septicemia (mice)	[279]
*Yersinia enterocolitica*	Acylation of lipid A	*lpxP* and *htrB*	Polymyxin B	Gastrointestinal infection (mice)	[280]
	OMP-mediated protection	*yadA* (pYVe plasmid-encoded)	Lysozyme and defensins from human granulocytes	Adhesion, autoaggregation, resistance to complement-mediated killing	[281]
	Active efflux (MFS-type)	*rosAB*	Polymyxin B, cecropin, and melittin	Unknown	[282]
*Yersinia pestis*	Degradation by aspartate protease	*pla*	LL-37, rCRAMP, and rat β-defensin-1	Plague (mice)	[130]
	Active efflux (RND-type)	*acrAB*	Polymyxin B	Not required	[283]
*Yersinia* *pseudotuberculosis*	Acylation of lipid A	*pagP*	Polymyxin B and cecropin	Unknown	[284]

* Roles in virulence and pathogenicity have been demonstrated in animal models, in vitro cell cultures and bioassays, and ex-vivo studies, either performed within the same study or independently. These roles may not conclusively be linked to antimicrobial peptide (AMP) resistance per se. The underlined genes are regulatory genes. Abbreviations: BMAP-28: bovine myeloid antimicrobial peptide (cathelicidin family), Blebs: vesicles containing DNA, LOS, and OMPs; BPI: bactericidal/permeability-increasing Protein; cBD-1: caveolin-1-binding domain; CRAMP: cathelicidin-related antimicrobial peptide C18G: synthetic α-helical peptide derived from human platelet factor I; ETEC: enterotoxigenic Escherichia coli; EPSs: extracellular polymeric substances; HBD: human β-defensin; HNP: human neutrophil peptide; LBP-B: lactoferrin-binding protein B; LPS: lipopolysaccharide; LOS: lipooligosaccharide; mCRAMP: murine cathelicidin; OM: outer membrane; OMP: outer membrane protein; P-113: AMP derived from the human salivary protein histatin 5; P2 and P6: bioactive peptide fragments; PC-8: linearized synthetic variant lacking both disulfide bonds; PG-1: protegrin-1 (porcine); PR-39: a proline-rich antibacterial peptide; RIA and RIB: enzymes arising by the differential processing of the prpR1 arginine-specific protease; rCRAMP: rat cathelicidin.

**Table 3:** Mechanisms of AMP resistance in Gram-positive bacteria and their correlation with virulence and pathogenicity.

Bacteria	Mechanisms/Regulatory Pathways of Resistance	Gene(s) Involved	AMPs	Correlation with Pathogenicity/Virulence *	Refs.
*Bacillus anthracis*	Degradation by metalloproteases	*clpX*	LL-37, α-defensins, and lysozyme	Lethal infection in CRAMP ^−/−^ mice	[285,286]
	Capsule-mediated protection	*capA*	Defensins, gramicidin, polymyxin B, nisin, protegrin, and melittin	Dissemination of inhalation anthrax infection (guinea pig)	[25,287,288]
	D-alanylation of TAs	*dlt*	Polymyxin B, colistin, nisin, and magainin-2	Survival in macrophages and full virulence in a mouse model of inhalational infection	[289]
	Lysinylation of PG	*mprF*	Protamine, LL-37, and HNP-1	Unknown	[290]
*Bacillus cereus*	D-alanylation of TAs	*dlt*	Protamine, nisin, polymyxin B, colistin, lysozyme, and cecropin B	Septecaemia and virulence (insects)	[291]
	Proteolytic degradation by zinc metalloproteases	*inhA1* and *inhA2*	Cecropin and attacin	Escape from host macrophages Lethal infection by injection into insects	[292,293]
*Bacillus subtilis*	D-alanylation of TAs	*dlt* (*spoO* and *abrB*)	Nisin	Unknown	[294,295]
	Alteration of cytoplasmic membrane lipid composition	*sigX*	Nisin	Unknown	[294]
*Clostridium difficile*	D-alanylation of TAs	*dlt*	Nisin, polymyxin B, and gallidermin	Unknown	[296]
*Enterococcus faecalis*	Degradation by gelatinase	*gelE*	LL-37 and HYL-20	Peritonitis (mice)	[240,297]
	Degradation by serine proteases	*sprE*	HYL-20	Peritonitis (mice)	[298,299]
	Shedding of host proteoglycans and neutralization of AMPs	Undefined (probably *gelE*)	Neutrophil-derived α-defensins	Unknown	[300]
	D-alanylation of TAs	*dlt*	Colistin, nisin, and polymyxin B	Unknown	[301]
	Lysinylation of phospholipids	*mprF1* and *mprF2*	Defensins and daptomycin	Bacteremia (mice)	[302,303,304]
	Alteration of the localization of cardiolipin microdomains	*liaR*	Daptomycin and telavancin	Unknown	[305]
	O-acetylation of peptidoglycan	EF_0783	Lysozyme	Survival in peritoneal macrophages (mice)	[306]
Group A streptococcus	Degradation by cysteine-proteinases	*speB*/*ideS* (*covRS* also known as *csrRS*)	LL-37	SpeB highly expressed in vivo and colocalizes with LL-37 in human tissue samples	[240,307]
	Capsule (hyaluronic acid)-mediated repelling	*hasABC* (*covRS)*	LL-37	Survival in neutrophil extracellular traps	[308]
	Secreted and surface-bound inhibitory proteins	*emm1* (Fimbrial M1 proteins)	LL-37	Skin or systemic infection (mice)	[309]
		*ska* streptokinase	LL-37 and other cationic AMPs	Systemic dissemination and virulence (mice)	[310,311]
		*sic*	LL-37 and defensins	Skin infection (mice)	[312]
	Shedding of host proteoglycans that bind cationic AMPs	*lasA* and *speB*	LL-37 and defensins	Skin infection (mice)	[300,313]
	Cleavage by GRAB:SpeB complex	*speB* *grab*	LL-37	Skin infection (mice)	[314]
	Regulatory systems sensing and inducing AMP resistance	*covRS*	LL-37	In vivo induction by LL-37	[315,316]
		*crgR*	mCRAMP	Competitive advantage (mice)	[317]
	D-alanylation of TAs	*dlt*	LL-37, polymyxin B, and lysozyme	Resistance to neutrophil killing, adhesion, and invasion (pharyngeal epithelial cells)	[318]
	Active efflux (ABC-type)	*salY*	SalA and SalA1 lantibiotics	Intramacrophage survival (zebrafish)	[319]
	Manipulation of host AMP production	Unknown	Defensins	Unknown	[320]
Group B streptococcus	TCS regulatory pathways	*liaR* and *covRS*	Polymyxin B, colistin, nisin, and LL-37	Sepsis and pneumonia (mice)	[321,322]
		*ciaR*	mCRAMP and lyzozyme	Intracellular survival within neutrophils, murine macrophages, and human brain microvascular endothelial cells	[322]
	Competitive binding/inactivation by PBP1a	*ponA* (*liar*)	LL-37, CRAMP, and defensins	Antiphacocytic Pulmonary infection and sepsis (rats)	[323,324]
	Sequestration by pili	*pilB* (*liar*)	LL-37 and mCRAMP	Invasion and paracellular translocation mediating resistance to phagocytic killing and virulence (humans and animal models)	[325,326]
	D-alanylation of TAs	*dlt* (*dltSR*)	Colistin	Pulmonary or systemic infection (rats)	[327,328]
*Listeria* *monocytogenes*	N-deacetylation of peptidoglycan	*pgdA*	Lysozyme	Virulence after oral and IV inoculations (mice) Survival in macrophages, liver, spleen, and intestinal lumen	[329]
	O-acetylation of peptidoglycan	*oat*	Lysozyme	Virulence, survival in macrophages, and control of cytokine production (mice)	[330]
	Glycosylation of WTAs	*gttA, gltB,* and *rml*	LL-37	Intestinal epithelium colonization (mice)	[331,332]
	D-alanylation of TAs	*dlt* (*virR*)	Colistin, polymyxin B, and nisin	Blood infection (mice)	[333,334]
	Lysinylation of PG	*mprF* (*virR*)	Gallidermin, HNP-1 and -3	Survival in liver and spleen (mice)	[334,335]
	Active efflux (ABC-type)	*anrAB* (*virR* and *rpoN*)	Nisin, bacitracin, and gallidermin	Unknown	[336]
	Thermoregulated transcription factor	*prfA*	Potato defensin (thermo-dependent)	Gastrointestinal infection (mice)	[337]
*Mycobacterium* *tuberculosis*	Lysinylation of PG	*lysX*	HNP-1 and lysozyme	Respiratory infection (mice and pig)	[338]
*Mycobacterium marinum*	Mycolic acid-mediated protection	*kasB*	HNP-1 and lysozyme	Intramacrophage survival	[339]
*Staphylococcus* *aureus*	Degradation by metalloprotease aureolysin	*aur*	Haloganan and LL-37	Not required	[340,341,342]
	Degradation by V8 glutamylendopeptidase (serine protease)	*sspA*	LL-37	Virulence and in vivo growth (murine abscess models)	[341,343,344]
	Inhibition by iron regulated surface determinant A	*isdA*	Lactoferrin and LL-37	Initial stage of abscess formation after IV infection (mice)	[345,346,347]
	Inhibition by staphylokinase (plasminogen activator protein)	*Sak* (*agr*)	Lactoferricin, tritrpticin, and human defensins	Establishment of skin infections (humans and mice models)	[348,349,350,351]
	O-acetylation of peptidoglycan	*oat*	Host lysozyme	Septic arthritis (mice) Anti-inflammation Inhibits the polarization of T-helper cells (mice)	[352,353,354,355,356,357]
	WTA-mediated protection	*tagO*	Group IIA phospholipase A2 and HBD-3	Induction and progression of endovascular infection (rabbit model of infective endocarditis), adherence to human epithelial cells biofilm formation, colony spreading, and virulence in mammals	[358,359,360,361,362,363]
	D-alanylation of TAs	*dlt* (*agr*)	HNP1–3, gallidermin, protegrins 3 and 5, tachyplesins-1 and 3, magainin-2, nisin, and tPMP-131	Sepsis and septic arthritis (mice)	[364,365,366,367,368,369]
	Alteration of cytoplasmic membrane lipid composition	*pgsA* and *cls2*	Daptomycin	Unknown	[370]
	Lysinylation of PG	*mprF/lysS*	Defensins	Systemic infection (mice)	[371,372,373]
	Active efflux (ABC-type)	*pmtABCD*	Defensins and LL-37	Skin infection (mice)	[374]
	Plasmid-mediated active efflux (MFS)	*qacA*	tPMP	Endovascular infections (rabbit)	[375]
	TCS inducing AMP resistance	*graRS *and *apsSX/apsR*	HBD3, nisin, indolicidin, and LL-37	Kidney infections (peritoneal infection murine model)	[367,368,376]
	Active efflux (ABC-type)	*vraFG*	HBD3, nisin, indolicidin, and LL-37	Hemolytic activity, expansion of subcutaneous abscesses	[368,376,377]
*Staphylococcus* *epidermidis*	Sequestration/inhibition by EPS: PIA also known as PNAG	*ica*	HBD-3, LL-37, and anionic dermcidin	Resistance to PMN killing (humans)	[378]
	AMP-inducible three component systems	*apsXRS * (via *dlt*, *mprF*, and *vraFG*)	HBD3	Resistance to PMN killing (humans)	[369,379]
*Staphylococcus* *xylosus*	D-alanylation of TAs	*dlt*	Gallidermin, magainin 2, and nisin	Unknown	[365]
*Streptococcus iniae*	Shielding/ inhibition by polyanionic surface capsule and cell wall structures	*pgm* (phosphoglucomutase)	Moronecidin and mCRAMP	Meningoencephalitis (hybrid striped bass model)	[380]
	O-acetylation of peptidoglycan	*cpsY *and *oatA* (*metR/mtaR*)	Lysozyme	Survival in neutrophils	[381]
*Streptococcus* *mutans*	Active efflux (ABC-type)	*bceABRS* (formerly *mbrABCD*)	Bacitracin and human α- or β-defensins (also induce *bce*)	Unknown	[382]
	D-alanylation of TAs (planktonic cells and biofilm)	*dlt* (*ciaR*)	HBD-1, HBD-2, HBD-3, and LL37	Regulation of cariogenic virulence	[383,384]
*Streptococcus* *pneumoniae*	Inhibition by pneumococcal surface protein A	*pspA*	Apolactoferrin	Pneumococcal infection (mice)	[385]
	Neutralization by free anionic capsular polysaccharide	*cps*	Polymyxin B and HNP-1	Unknown	[241]
	D-alanylation of TAs	*dlt* (*ciaRH*)	Nisin and gallidermin	Competitive advantage in murine model of pneumococcal pneumonia	[386,387,388]
	Active efflux (ABC-type)	*macAB* homolog	Bacitracin, LL-37, and nisin	Unknown	[389,390]
	Active efflux (MFS-type)	*mefE*	Defensins, LL-37, and CRAMP	Unknown	[389,391]
	N and O-acetylations of peptidoglycan	*pgdA* and *adr*	Lysozyme	Colonization of the upper respiratory tract (mice)	[392]
*Streptococcus suis*	Degradation by cysteine protease	*apdS*	LL-37	Meningitis and sepsis (humans)	[393]
	O-acetylation of peptidoglycan	*oat*	Lysozyme	Unknown	[394]

* Roles in virulence and pathogenicity have been demonstrated in animal models, in vitro cell cultures and bioassays, and ex-vivo studies, either performed within the same study or independently. These roles may not conclusively be linked to antimicrobial peptide (AMP) resistance per se. The underlined genes are regulatory genes. Abbreviations: CRAMP: cathelicidin-related antimicrobial peptide; ETEC: enterotoxigenic Escherichia coli; EPSs: extracellular polymeric substances; GRAB: α2-macroglobulin/G-related α2-macroglobulin-binding protein; HBD: human β-defensin; HNP: human neutrophil peptide; HYL-20: peptide derived from the venom of the solitary bee *Hylaeus signatus*; mCRAMP: murine cathelicidin; PBP1a: penicillin-binding protein 1a; PG: phosphatidylglycerol; PIA: polysaccharide intercellular adhesin also known as Poly N-acetylglucosamine (PNAG); TA: teichoic acid; WTA: wall teichoic acid.

### 3.2. Extracellular Trapping and Inactivation

Given the limited efficacy of proteases against defensins and other AMPs, numerous bacterial pathogens have devised supplementary strategies to impede their function (Table 2 and Table 3). Many of these tactics are tailored to specific peptides, encompassing processes such as recognition and extracellular capture. The direct capture or neutralization of AMPs can occur through the action of secreted proteins or those affiliated with the bacterial surface, while indirect methods involve host cells releasing surface molecules capable of binding PAMs (Table 2 and Table 3). Consequently, when a substantial portion of AMPs released during the innate immune response hampers these molecules, inadequate quantities reach the bacterial cell membrane.

#### 3.2.1. AMP-Inhibitory Proteins Associated with the Bacterial Cell Surface

The sequestering of AMPs by certain molecules prevent them from interacting with their targets (Figure 5C). For example, SGA generate fimbrial M proteins, which steadfastly adhere to the cell wall, enveloping themselves in mucoid layers. Lauth et al. (2004) revealed that virulent strains producing the M1 serotype of this protein display a notably heightened resistance to cathelicidin LL-37 compared to their less virulent counterparts (housing M proteins of alternative serotypes) [33]. This protein is capable of binding to the cathelicidin precursor (hCAP-18), inhibiting its transformation into a mature AMP, and it can also bind to LL-37, thereby evading its effect [395].

Streptococci and staphylococci produce a variety of surface proteins able to degrade cationic AMPs (Table 3). For example, *S. aureus* expresses the iron-regulated surface determinant A (IsdA), located on the peptidoglycan layer, which is responsible for resistance to lactoferrin and mCRAMP. This adhesin, highly expressed during iron deficiency, confers resistance to AMPs via its C-terminal domain, which binds to apolactoferrin and suppresses its protease activity. Notably, IsdA does not necessitate attachment to the bacterial surface to manifest its proteolytic activity [345,346].

*Neisseria gonorrhoeae* and *N. meningitidis* produce a membrane-bound lipoprotein known as lactoferrin-binding protein B (LbpB) (Table 2). This protein contains anionic domains capable of conferring resistance to lactoferricin B, derived from host lactoferrin, an iron-sequestering antimicrobial protein released by neutrophils [217,218]. Recent structural studies have revealed that the N-lobe of LbpB harbors the binding site for lactoferrin. While LbpB may not be essential for iron acquisition in vivo, it plays a crucial role in protecting against cationic AMPs when membrane-bound. The enzymatic release of *N. meningitidis* LbpB from the cell surface by the bacterial protease NalP is hypothesized to contribute to LbpB’s function as a “Cationic AMP sink” [225,396]. Studies have also shown that the anionic regions of LbpB from *Moraxella catarrhalis* protect bacteria against cationic AMPs [397]. Additionally, LbpB-mediated protection against cationic AMPs is highly specific, as it provides resistance to the mCRAMP and certain synthetic peptides (IDR-1002 and IDR-0018) but not others (Tritrp 1 or LL37, HH-2, or HHC10) [397]. Recently, Ostan et al. (2024) demonstrated that the addition of lactoferricin to the anionic domain of LbpB or full-length LbpB secreted from the cattle pathogen *Moraxella bovis*, which expresses the largest anionic domain of the LbpB homologs, results in the formation of phase-separated droplets of LbpB together with the AMP. The droplets displayed a low rate of diffusion, suggesting that cationic AMPs become trapped inside and are no longer able to kill bacteria. Authors suggested that pathogens, like *M. bovis*, leverage anionic intrinsically disordered domains for the broad recognition and neutralization of antimicrobials via the formation of biomolecular condensates [398] (Figure 5C).

Finally, *N. gonorrhoeae* expressing type IV pili demonstrates resistance against LL-37 and neutrophil-mediated killing. However, non-piliated strains exhibit heightened susceptibility, potentially due to elevated intracellular iron levels. Iron chelation effectively protects non-piliated mutants from hydrogen peroxide and LL-37-mediated killing, indicating a role for iron availability in modulating bacterial sensitivity [219]. Therefore, the above-mentioned observations emphasize a critical relationship between piliation status, iron homeostasis, and susceptibility to host-derived AMPs (Figure 5E).

#### 3.2.2. AMP-Inhibitory Proteins Secreted by Bacteria

*S. aureus* secretes a plasminogen activator protein called staphylokinase (Sak), which has the ability to bind to certain AMPs and inhibit their activities. For example, Sak, especially the truncated Sak with its first ten residues, exhibits a lower affinity for melittin and mCRAMP, but shows a high affinity for lactoferricin and tritrpticin [350]. In addition, Sak can bind to human β-defensins and restrict their bactericidal activity [349]. Similarly, GAS hijacks host plasmin through the secreted plasminogen activator streptokinase (Ska) to evade the lethal effects of cathelicidin, facilitating their evasion from innate immunity [310]. Moreover, the virulent strains of GAS M1 secrete a protein known as SIC (Streptococcal Inhibitor of Complement), which is absent in the vast majority of non-invasive GAS strains. SIC is capable of binding to and inactivating LL-37 and human α-defensin [312] (Table 3) (Figure 5D).

#### 3.2.3. AMP-Inhibitory Molecules Released by Host Tissues

Bacteria appear to trap and deactivate PAMs by exploiting the negatively charged proteoglycan molecules that decorate the surfaces of host epithelial cells. For instance, extracellular proteases, such as cysteine proteinases, gelatinases, elastases, and alkaline proteinases, secreted by bacteria such as *S. pyogenes*, *E. faecalis*, and *Pseudomonas aeruginosa*, respectively, degrade human cell surface proteoglycans, such as decorin, then releasing dermatan sulfate (Table 2 and Table 3). The released dermatan sulfate binds to and inactivates human α-defensins [300]. In addition, *P. aeruginosa* produces elastase LasA, which increases the release of syndecan-1, a cell-surface heparan sulphate proteoglycan, from host cells. Consequently, syndecan-1 binds to and inactivate cationic AMPs [242] (Table 2 and Table 3).

### 3.3. Electrostatic Shielding/Sequestration of AMPs by the Capsule

The production of capsule polysaccharides (CPSs) is involved in adjusting biofilm formation to cause persistent infections in the blood, respiratory tract, and gastrointestinal mucosa of mammals [399,400]. Additionally, it can either confer resistance or reduce susceptibility to several AMPs and evade phagocytosis [401]. While the chemical composition of CPSs varies widely among bacterial species, the majority of them are anionic. The interaction with the bacterial capsule is believed to trigger conformational changes that lead to the sequestration of AMPs, consequently inhibiting their capacity to reach their target on the bacterial membrane [402].

The role of CPSs as a physical barrier in resistance to AMPs has been extensively studied, as observed in *K. pneumoniae*, *S. pyogenes* TX72 [204], and *N. meningitidis* [226], whose CPS mutants display increased sensitivity to AMPs compared to wild-type (WT) strains (Table 2 and Table 3). Furthermore, the degree of *K. pneumoniae* resistance to polymyxin B and lactoferrin is proportional to the amount of CPSs produced as a result of *cps* gene induction by these two AMPs [204]. Purified CPSs from *K. pneumoniae*, *S. pneumoniae*, and *P. aeruginosa* effectively sequester cationic AMPs and neutralize their bactericidal activity. The release of CPSs into the milieu increases significantly when bacteria are exposed to polymyxin B and human neutrophil peptide-1 (HNP-1) [241]. Additionally, O’Brien et al. elucidated that non-capsulated *Bacillus anthracis* strains are markedly more susceptible to various AMPs, compared to their encapsulated counterparts [25] (Table 3) (Figure 5F).

### 3.4. AMP Resistance Mechanisms Associated with Bacterial Cell Wall Structures and Their Modifications

The bacterial cell wall, primarily composed of peptidoglycan, is an important target for several antimicrobial agents. It is found outside the bacterial cell membrane, serves as a protective barrier against environmental stresses, and reinforces cell shapes [403]. AMPs face the initial challenge of penetrating the bacterial cell envelopes, which varies between Gram-negative and Gram-positive bacteria. In Gram-negative bacteria, this barrier includes an outer membrane in addition to a thin peptidoglycan layer and the cytoplasmic membrane, with the outer membrane being composed of phospholipids, lipopolysaccharides (LPSs), and proteins. Conversely, Gram-positive bacteria lack an outer membrane but possess a thicker peptidoglycan layer to which teichoic acids (TAs) and lipoteichoic acids (LTAs) are anchored [404]. Hence, most bacterial resistance mechanisms operate by altering the chemical components of these surface structures to restrict the interaction with AMPs.

#### 3.4.1. Involvement of LPS and Its Modifications in Resistance to AMPs

The outer membrane plays a crucial role in the Gram-negative bacterial natural defense by exhibiting reduced permeability, and regulating its permeability and integrity is pivotal [405,406]. The principal component of the OM is the negatively charged LPS, which attracts cationic AMPs, while outer membrane proteins (OMPs) also contribute to this resistance through their physiological roles, as discussed in Section 3.4.2. While bacterial plasma membranes share the same phospholipid composition, certain AMPs exhibit effectiveness against specific bacterial strains but not others [407]. This difference in activity highlights variations in the structure and composition of LPS among bacterial species, which consequently affect the barrier properties of the outer membrane permeability. The LPS consists of three components: lipid A, core oligosaccharide, and the O-polysaccharide or O antigen (Figure 3). It is highly polyanionic, primarily due to (i) the presence of two monophosphate groups substituting the lipid A at positions 1 and 4′, and (ii) the carboxylic groups of the 3-deoxy-D-manno-oct-2-ulosonic acid (Kdo), along with monophosphate (or pyrophosphate or diphosphate) groups substituting the heptose residue(s) at the inner core level. Additionally, the oligosaccharide core harbors numerous anionic groups, the distribution of which varies among Gram-negative bacterial species [405]. The O-antigen composition varies significantly and is unique to each bacterium [185,408]. The enzymes responsible for catalyzing the biosynthesis of phospholipids and LPS are well characterized in *E. coli* and are located either in the cytoplasm or on the inner cytoplasmic leaflet of the inner membrane [409,410]. Considering the crucial role of LPS in protecting bacteria from the entry of hydrophobic and hydrophilic molecules [411], enzymes involved in LPS biosynthesis, such as Lpx [206], and LPS modifications induced by PhoPQ and PmrAB, play a significant role in the resistance of many Gram-negative bacteria against AMPs [216,412] (Table 2). Additionally, specific mutations within these TCSs, as well as in ColRS, ParRS, and CprS TCSs of *P. aeruginosa*, lead to their constitutive activation, resulting in the subsequent overexpression of LPS-modifying genes in these bacteria [37].

##### Resistance to AMPs Due to O-Antigen and Core Oligosaccharide

The specific O chains define the surface properties of bacteria in species that do not produce capsules. It acts as a protective shield that prevents the entry of AMPs into the LPS bilayer (Table 2). These oligosaccharide chains are crucial for the bacteria that produce them to avoid recognition by the host’s immune defenses. For example, *C. jejuni* mimics the gangliosides (glycolipids) on the surface of its host’s eukaryotic cells [413]. O-antigens have been shown to provide protection to *S. flexneri* and *K. pneumoniae* by shielding them from histones, significant components of neutrophil extracellular traps and acting as AMPs [205]. In addition, a larger O-antigen provides increased protection against AMPs. Indeed, resistance to AMPs is significantly reduced in several bacteria that possess truncated or completely absent LPS antigens, commonly referred to as rough LPS mutants (LPS-R), compared to WT bacteria with smooth LPS (LPS-S) [239,414,415,416,417,418] (Table 2).

Such differences in susceptibility are most likely due to variations in the LPS content of divalent cations [419,420]. In fact, LPS chelates a multitude of divalent cations, including Mg^2+^ and Ca^2+^, owing to its negative charges [421,422,423]. The partial neutralization of these negative charges by the bridges formed with these cations diminishes repulsion between adjacent LPS molecules and stabilizes lateral interactions [424]. AMPs interacting with the outer layer of LPS competitively displace divalent cations, causing the destabilization of the LPS bilayer [425]. Interactions between neighboring O chains may thus contribute to the stability of this bilayer [426]. These intermolecular interactions through O chains (and likely the outer core) are markedly more significant in LPS-S than in LPS-R [427]. Yeh and Jacobs (1992) demonstrated that the presence of long O chains in LPS reduces the fluidity of the phospholipid bilayer more effectively than those with short or no O chains. This indicates that O chains influence the behavior of the lipid A domain [428].

The structure of LPS can influence the interaction of AMPs with the outer membrane. This has been elucidated in *S.* Typhimurium with magainin 2, showing that the sensitivity of Gram-negative bacteria to this peptide is associated with factors that facilitate the transport of AMPs across the outer membrane. These include the importance and location of LPS charge, LPS concentration in the outer membrane, its architecture, as well as the presence or absence of the lateral chain of the O-antigen [429]. Several studies have subsequently shown that resistance to AMPs depends on variations in the chemical composition of LPS, such as the importance of the charge of O antigen saccharide chains and the specific glycosylation of the latter (through *gtr* genes). Glycosylation leads to a modification of the conformation of the O antigen subunits by halving the thickness of the LPS layer, which can increase bacterial resistance to AMPs, particularly in *S. flexneri* and *Bordetella* sp. [151,272].

The enzymes involved in the biosynthesis of the outer and/or inner core oligosaccharide, the nature of the latter, and the density of the O-polysaccharide chain all contribute, either collectively or individually, to enhanced resistance to AMPs (Table 2). For example, the removal of this core can downregulate the expression of the *pmrD* gene responsible for *E. coli* resistance against polymyxins [179]. In *C. jejuni*, the *galU* gene is indeed responsible for synthesizing uridine diphosphate (UDP)-glucose, which is a precursor molecule required for the synthesis of LPS. When the *galU* gene is mutated or disrupted, the production of UDP-glucose is impaired, resulting in the truncation of the LPS core structure. This truncation makes the bacterium more susceptible to AMPs and other antimicrobial agents [164].

##### Roles of the Acylation and Modifications of Lipid A Acyl Chains in Intrinsic and Induced Resistance to AMPs

The higher content of saturated fatty acids in LPS, coupled with divalent cations, induces rigidity in LPS [419,430,431]. Several experiments have conclusively shown that the portion of LPS anchored in the outer membrane, specifically the fatty acids, exhibits a remarkably low fluidity [432,433]. Fluidity, in this context, refers to the ability of molecules to move within the same layer. Unlike PG, the glycerophospholipids typically found in other biological membranes such as the cytoplasmic membrane, which contains only two chains of saturated fatty acids, LPS comprises between four and seven chains. However, unlike unsaturated fatty acids, saturated fatty acids reduce the fluidity of the lipid layer they compose due to their larger steric hindrance [434]. Consequently, the absence of unsaturated fatty acids renders the inner leaflet of the LPS (comprising fatty acid chains) much less fluid, thereby diminishing the permeability of the outer membrane. Furthermore, the number, distribution, length, and type of the acyl chain in the fatty acid of lipid A vary among various bacteria, such as *Acinetobacter baumannii*, *B. cepacia*, *B. pseudomallei*, *C. jejuni*, *H. pylori*, *E. coli*, *N. gonorrhoeae*, *K. pneumoniae*, *S. enterica*, *P. aeruginosa*, *Vibrio cholerae*, and *Y. pestis*, which can influence their membrane properties [435].

Acylation of Lipid A in LPS

Lipid A typically contains four primary chains of (R)-3-hydroxyacyl directly and covalently linked to the glucosamine disaccharide at positions 2, 3, 2′, and 3′. Some of these (R)-3-hydroxyacyl groups are modified by secondary acyl chains, forming acyloxyacyl fractions [435]. For example, in *E. coli* and *S*. Typhimurium, secondary acyl groups of laurate (12 carbons) and myristate (14 carbons) are attached to the 3-Kdo_2_-lipid A at positions 2′ and 3′ of the primary acyl chain, respectively (Figure 3). The incorporation of lauroyl chain is mediated by the LpxL acyltransferases (HtrB in *Salmonella*, *Shigella*, and *H. influenzae*), while the myristoyl chain is mediated by LpxM (formerly MsbB or WaaN) [435,436]. The activation of HtrB requires a high temperature in *E. coli* [437], whose genome also contains the *lpxP* gene (homologous to *lpxL*), induced by cold shock (12 °C). LpxP catalyzes the incorporation of palmitoleate (unsaturated fatty acid) instead of laurate, suggesting the bacterium needs to adjust the fluidity of its outer membrane [436,438].

Only a limited number of laboratory knockout mutations (through insertional inactivation) can be generated at the lipid A level without impacting bacterial viability. Among these mutations are those affecting the *lpxA*, *lpxM*, and *htrB* genes. For instance, in *N. meningitidis*, a viable *lpxA* (encoding UDP-GlcNAc acyltransferase required for the first step of lipid A biosynthesis) mutant that is entirely deficient in LPS experiences severe growth impairment. Additionally, there is an increase in phosphatidylethanolamine (PE) and PG species, predominantly composed of shorter chains, thus hindering in vivo analyses [439]. On the other hand, the inactivation of *lpxM* has been extensively studied in several Enterobacterales [440,441]. Myristoylation of lipooligosaccharide (LOS), catalyzed by the acyltransferase HtrB, is a crucial factor in conferring resistance to human β-defensin by the human pathogen *H. influenzae*. Mutant strains lacking HtrB are 45 times more sensitive than the WT [198]. However, mutations in the *lpxM* gene in several bacterial pathogens do not provide conclusive findings regarding the resulting phenotypes. For instance, *lpxM* mutants of *S.* Typhimurium and *S. flexneri* exhibit attenuated ability to induce inflammation in vivo [440,442]. Alterations in growth and membrane permeability observed in the *lpxM* mutants of *S.* Typhimurium lead to the selection of extragenic suppressor mutations in this bacterium [443]. The only instance of observed virulence attenuation in a murine infection model directly linked to the *lpxM* gene mutation was observed in a clinical strain of *E. coli*. The *lpxM* gene is not indispensable for *E. coli* growth [443,444,445], but its mutation leads to side effects such as capsule reduction [446]. Furthermore, the inactivation of the *lpxP* gene in *E. coli* does not affect bacterial growth but heightens bacterial sensitivity to certain antibiotics at low growth temperatures [447]. The role of covalent modifications catalyzed by LpxM in maintaining membrane integrity, as well as enhancing its permeability and resistance to cationic AMPs, has also been elucidated in *K. pneumoniae, A. baumannii,* and *V. cholerae* [206] (Table 2).

Resistance to polymyxins in *E. coli* and *S.* Typhimurium requires the LpxM-mediated myristoylation of their lipid A since the addition of L-Ara4N to the phosphate groups of lipid A (catalyzed by l- Ara4N transferase ArnT) depends on the presence of the secondary myristate (catalyzed by LpxM) chain at position 3′ of glucosamine [182]. Moreover, mutations of *lpxP* and *htrB* in *Y. pestis* result in increased susceptibility to polymyxin B at 21 °C and 37 °C, respectively. Additionally, the acylation of lipid A plays a role in the expression of virulence factors [280].

*A. baumannii* can develop resistance to colistin through the loss of its LPS (Table 2). The outer membranes of polymyxin-resistant strains, with lipid A modified with PEtN or deficient LPS, exhibit distinct atomic-level interactions with polymyxins [448]. In mouse models, LPS-deficient *A. baumannii* strains exhibit reduced virulence. The development of colistin-resistant LPS-deficient strains involves spontaneous mutations in genes such as *lpxA*, *lpxC* (encoding UDP-3-O-(R-3-hydroxymyristoyl)-N-acetylglucosamine deacetylase), and *lpxD* (encoding UDP-3-O-(3-hydroxymyristoyl) glucosamine N-acetyltransferase). Studies have shown that LPS-deficient *A. baumannii* weakly stimulate neutrophils, leading to lower levels of reactive oxygen species (ROS) and inflammatory cytokine production. Furthermore, LPS-deficient *A. baumannii* strains are more susceptible to antibacterial lysozyme and lactoferrin compared to their WT counterparts [133,135]. Jennifer H. Moffatt et al. (2013) also demonstrated that LPS-deficient *A. baumannii* are more susceptible to LL-37 and exhibit altered signaling through host Toll-like receptors [134].

Some *Francisella* species, like *F. tularensis*, responsible for tularemia, synthesize relatively short LPS molecules with a lipid A devoid of Kdo and other saccharide chains [449]. The lipid A of these bacteria is devoid of both a phosphate group and acyl chains at the 4′ and 3′ ends, respectively. The dephosphorylation of lipid A in *Francisella* is facilitated by the 4′- phosphatase LpxF, a constituent of the LPS biosynthesis pathway in this bacterium. The *lpxF* mutant of *F. novicida* displays a heightened sensitivity to cationic AMPs [193] (Table 2). This increased susceptibility damages the bacterial envelope, exposing membrane lipoproteins and bacterial DNA to Toll-like receptors TLR2 and TLR9, respectively [450]. Notably, the dephosphorylation of lipid A by LpxF is contingent upon the absence of a secondary myristate chain at position 3′ due to its steric hindrance chain near the 4′-phosphate group [193].

Similarly, *H. pylori*, *Rhizobium leguminosarum*, *P. gingivalis*, and *Rhizobium etli* tend to remove the anionic phosphate group from the lipid A to resist against AMPs by using the 4′-phosphatases LpxF [167]. On the other hand, some bacteria, such as *Helicobacter pylori*, has a tendency to remove the 1-phosphate group from lipid A using the 1-phosphatase LpxE encoded by *lpxE* gene, and subsequently add the phosphoethanolamine (PEtN) group, facilitated by the EptA enzyme [167,201,202]. Furthermore, the deletion mutants of *lpxE* gene in *Capnocytophaga canimorsus* and *Brucella abortus* exhibited increased polymyxin B sensitivity [155,167,245] (Table 2).

*pagP*, one of the genes induced by PhoPQ in *Salmonella*, encodes an unusual acetyltransferase named PagP, located in the outer membrane and involved in LPS biosynthesis. PagP catalyzes the transfer of palmitate from PE (donor) to the OH group of the 3-hydroxymyristate chain linked at position 2 to the lipid A of LPS (Figure 3). This increases the number of acyl groups per lipid A molecule to a total of seven chains (in addition to the six chains constitutively incorporated) [451]. Furthermore, the palmitate chain is longer than those previously incorporated during lipid A biosynthesis as it consists of 16 carbon atoms (C16:O). All these functions are likely to alter the fluidity of the outer membrane by increasing hydrophobic interactions and van der Waals bonds between the acyl chains of neighboring LPS molecules. This stabilization is suggested by the fact that palmitate incorporation catalyzed by PagP confers resistance to cationic AMPs in *S. enterica* [452] and attenuates the ability of LPS to trigger immune responses via the TLR4 Toll pathway [453]. PagP mutants of *S.* Typhimurium are sensitive to certain AMPs, such as C18G and protegrins, but are resistant to polymyxins. The increased acylation of lipid A alters the hydrophobic interaction between fatty acids and the AMP, thereby preventing or at least delaying its insertion into the bilayer [454]. The involvement of the *pagP* gene (or its homologues) in inducible resistance to AMPs has also been demonstrated in the pathogens *Bordetella parapertussis*, *B. bronchiseptica* (*rcp* gene for resistance to cationic antimicrobial peptides) [153], *Legionella pneumophila*, and *Yersinia pseudotuberculosis* [216,284,455]. The regulation of these genes is correlated with the bacterial lifestyle during infection. Some studies have highlighted that PagP might function as an apical sensory transducer, which can be activated by a breach in the outer membrane of the enterohemorrhagic strain *E. coli* O157:H7. Indeed, PagP is capable of sensing assaults that alter the permeability of the outer membrane, such as the action of EDTA, which chelates Mg^2+^ cations, or mutations affecting the presentation of LPS on the bacterial surface. Thus, the mutation of the *lpxM* (*msbB*) gene of *E. coli* O157:H7 triggers the palmitoylation of lipid A catalyzed by PagP, thereby restoring the permeability barrier role ensured by the outer membrane [180].

Recently, Sun et al. have revealed the role of Crrab TCS in upregulating the PagP-mediated palmitoylation of glycerophosphoglycerols and lipid A in *K. pneumoniae*, aiming to confer high-level polymyxin resistance and virulence. They demonstrated that PagP transfers palmitate from the glycerophospholipid of the OM inner layer to glycerophosphoglycerols, forming acyl-glycerophosphoglycerols, thereby boosting the ratio of acyl-glycerophosphoglycerols to glycerophosphoglycerols and enhancing outer membrane hydrophobicity [207].

Deacylation of Lipid A in LPS

*pagL* is a PhoPQ-governed gene encoding for PagL, an enzyme with lipase or deacylase activity, localized in the outer membrane of *S.* Typhimurium. It facilitates the deacylation or removal of the hydroxymyristate chain linked at position 3 to the glucosamine of lipid A in *Salmonella* [456] (Figure 3). Conversely, LpxR is another outer membrane lipase that cleaves the covalent bond between the acyloxyacyl chain linked at 3′ and the Kdo_2_-lipid A portion in a calcium-dependent manner [457]. PagL mutants of *S. enterica* are more sensitive to polymyxin B than the corresponding WT strain. Both LpxR and PagL lipases remain inactive in the outer membrane and are only activated upon overproduction, suggesting the presence of endogenous inhibitors, such as L-Ara4N [261]. The latency of PagL partly depends on the incorporation of PEtN at the Kdo_2_-lipid A level. The activation of PagL facilitates the deacylation of glucosamine at position 3, compensating for the decreased resistance to cationic AMPs caused by the absence of L-Ara4N at the lipid A of LPS [261]. Since this modification is only observed following PagL overproduction, it is possible to speculate that PagL is necessary for minor adjustments to the fluidity of the LPS layer after the addition of seven fatty acid chains to its lipid A.

Additionally, NaxD (*N*-acetylhexosamine deacetylase), a deacetylase from the YdjC superfamily, deacetylates N-acetylgalactosamine (linked to the lipid carrier undecaprenyl phosphate) to positively charged galactosamine, a necessary step for the subsequent addition of galactosamine to lipid A, contributing to polymyxin resistance in *Francisella* and *A. baumannii* [136,195] (Table 2). NaxD-mediated lipid A modification in *A. baumannii* is regulated by the sensor kinase PmrB [136]. Finally, spontaneous mutations and insertion sequences (IS) in the N-acetylglucosamine deacetylase encoding *lpxC* gene were shown to be associated with colistin resistance in colistin-resistant LPS-deficient strains of *A. baumannii* [133].

Hydroxylation of Lipid A in LPS

LpxO (or Pag Q) is an inner membrane Fe^2+^/alpha-ketoglutarate-dependent dioxygenase in *Salmonella* that catalyzes, in a PhoPQ-dependent manner, the hydroxylation (incorporation of the OH group) of the 2-hydroxymyristate chain of Kdo_2_-lipid A [458,459] (Figure 3). It compensates for the decrease in OH groups caused by PagP’s catalytic activity. The OH groups play a vital role as hydrogen bond donors, stabilizing interactions between neighboring LPS molecules. Unlike membrane phospholipids, the lipid A of LPS contains a number of hydroxylated fatty acids. Typically, the residues at positions 2 and 3 of glucosamine lack secondary myristate chains, resulting in two hydroxyl groups within the membrane that can act as hydrogen bond donors. The NH groups (2 groups at positions 2 and 2′) and the 4-OH group of reduced glucosamine are available to act as hydrogen bond donors. Additionally, the inner core oligosaccharide linked at position 6′ to the unreduced glucosamine provides numerous groups that could serve as both hydrogen bond donors and acceptors [405] (Figure 3). LpxO-dependent lipid A 2-hydroxylation has been shown to be essential for protecting *A. baumannii* against cationic AMPs, aiding its survival in human whole blood and the wax moth *Galleria mellonella* (Table 2). Additionally, LpxO shields *Acinetobacter* from *G. mellonella* AMPs, controlling their expression [137]. Similarly, in *K. pneumoniae*, LpxO-dependent modification dampens inflammatory responses and confers resistance to AMPs. Notably, a *lpxO* mutant exhibits attenuation in vivo, underscoring the significance of this lipid A alteration in *Klebsiella* infection. Colistin prompts the in vivo lipid A pattern, already expressed in colistin-resistant isolates, where LpxO-dependent lipid A modification is pivotal for resisting colistin [208]. Similarly, LpxO was suggested to confer resistance against AMPs and contribute to the pathogenicity of *S. enterica*, *P. aeruginosa*, and *B. bronchiseptica* [244,262,460]. Another enzyme called LpxN functions as a lipid A secondary hydroxy-acyltransferase in *V. cholerae*, responsible for the transfer of 3-hydroxylaurate to the lipid A domain significantly enhances polymyxin resistance [275] (Table 2).

##### Role of Cationic Polar Groups Added to Lipid A in Induced Resistance to AMPs

Cationic AMPs selectively bind to the negatively charged phosphate moieties and acidic groups within the lipid A and core polysaccharide by electrostatic interactions [419]. Many Gram-negative bacteria employ various strategies to reduce the interaction between cationic AMPs and their outer membranes by adding positively charged residues. The primary LPS modification involves the cationic substitution of phosphate groups with L-Ara4N, which neutralizes the net negative charge of lipid A, while the second most common modification is the addition of PEtN, reducing the net charge from −1.5 to −1 [136,405,461] (Figure 3). Among these modifications, the L-Ara4N substitution is deemed more effective due to its impact on charge alteration. For example, polymyxin-resistant Gram-negative species inherently possess LPS with one or more of these modifications. Indeed, *S.* Typhimurium emerges as a prominent example, highlighting how resistance to AMPs through alterations in LPS serves as a critical mechanism for survival. Although most of the genes required for these modifications are encoded in the chromosome, the recently identified plasmid-borne PEtN transferases (*mcr-*1 to *mcr*-10) pose a threat by potentially accelerating the spread of clinically significant colistin resistance [462].

Addition of Aminoarabinose to Lipid A in LPS

Gram-negative bacteria, including *E. coli* and *S. enterica,* reduce the negative charge of their surface by incorporating positively charged L-Ara4N at the 4′ phosphate end of their lipid A (Figure 3) (Table 2). This modification decreases the affinity of cationic AMPs, like polymyxin, to the outer membrane and reduces electrostatic repulsions between neighboring LPS molecules [463]. The addition of L-Ara4N occurs in a PhoPQ-dependent manner, which indirectly activates the PmrAB TCS via PmrD [183,464,465]. The biosynthesis and addition of L-Ara4N are catalyzed, respectively, by two genetic loci: the *pmrE* gene (also known as *pagA* or *ugd*) and the *pmrHFIJKLM* operon (also termed *arnBCADTEF* or *pbgP*) [466,467,468]. Non-polar mutagenesis studies have shown that all genes except *pmrM* are essential for the addition of L-Ara4N and resistance to cationic AMPs. A significant cause of colistin resistance in *K. pneumoniae* and *E. coli* involves mutations or the inactivation of the regulatory *mgrB* gene, which normally exerts a negative feedback on PhoPQ by inhibiting PhoQ kinase activity and/or enhancing its phosphatase activity. This results in the activation of PhoPQ, leading to the upregulation of all PhoP-mediated lipid A modifications, including the addition of L-Ara4N and PEtN to lipid A, as well as its deacylation [184,209]. Similarly, the PbgPE-mediated incorporation of 4-aminoarabinose into lipid A, in a PhoPQ-dependent manner, is essential for the resistance of the entomopathogen *P. laumondii* to polymyxins and insect-derived cecropins A and B, as well as for virulence in insects [233,234]. The substitution of lipid A moieties with L-Ara4N have also been demonstrated in vivo during cystic fibrosis caused by *P. aeruginosa* [247].

Addition of Phosphoethanolamine to Lipid A in LPS

The addition of PEtN to the LPS can counteract the effects of negatively charged residues. The PmrAB system controls the PEtN transferase EptA in *Salmonella* spp. and *E. coli* [185]. In *E. coli*, the PEtN addition to lipid A, Kdo, and heptose I of LPS is catalyzed by the EptA, EptB, and EptC enzymes, respectively. Another protein, termed CptA, responsible for the addition of PEtN to the core of *Salmonella* LPS, has been identified [264]. The incorporation of PEtN groups to lipid A and Kdo alters the net charge of LPS, providing defense against polymyxin B binding and penetration, while PEtN added to heptose I, alongside the reduction in negative charges, also prevents the insertion of the polymyxin B lipophilic tail into the outer membrane [186]. In *A. baumannii*, resistance to colistin is linked to the addition of PEtN to lipid A, facilitated by specific amino acid mutations in the PmrB protein. This results in the overexpression of *pmrC*, responsible for producing the PEtN transferase enzyme [138]. Recently, a novel TCS named StkSR was characterized in *A. baumannii*. Deleting *stkR* resulted in a notable upregulation of *pmrA*, *pmrC*, and *pmrB* expression, subsequently enhancing *pmrC* transcription and facilitating the replacement of lipid A with PEtN [139]. Another potential pathway that could result in the overproduction of PEtN in *A. baumannii* entails the integration of the IS*AbaI* insertion element upstream of an *eptA* isoform [140].

Further studies have demonstrated that constitutive mutations in the *pmrA* gene suppressed *msbB* mutant growth defects in *Salmonella*. In addition, the PEtN addition to lipid A confers polymyxin resistance in *msbB*-mutant strains, highlighting the necessity of aminoarabinose biosynthetic enzyme for the incorporation of PEtN and palmitate to the lipid A of these mutants [469].

#### 3.4.2. Outer Membrane Proteins and Resistance to AMPs

The outer membrane proteins (OMPs) of Gram-negative bacteria comprise a diverse array of proteins, including, among others, porins and adhesins, which fulfill diverse roles, such as signaling, adhesion, catalyzing reactions, and the active and passive transport of solutes and nutrients into and out of the cell [470]. AMPs have been proposed to utilize OMPs as a gateway for entry into bacterial cells [471]. This notion was supported by the observation that the absence of the MtrE channel unexpectedly increased gonococcal survival after exposure to azurocidin, prompting speculation about whether MtrE might facilitate the passage of certain antimicrobials across the outer membrane [224]. Additionally, the deletion of *tolC* has been found to enhance the activity of the transcriptional regulators MarA, SoxS, and Rob, which regulate porin expression and could potentially modify membrane permeability [472]. The idea that MtrE could act as a portal for AMP entry gained support from a recent discovery demonstrating that TolC is capable of importing bacteriocins (MW 60 kDa) in Gram-negative bacteria [473].

On the other hand, Visser et al. (1996) were among the first to describe the role of *Y. enterocolitica* adhesin A (YadA), in bacterial resistance to cationic AMPs [281]. In *V. cholerae*, the outer membrane proteins OmpU and OmpT, whose expression depends on the bacterial growth phase, partially contribute to basal resistance to cationic AMPs [276]. However, this resistance is further enhanced in the presence of AMPs in the bacterial environment. OmpU appears to act as a membrane sensor that detects antimicrobial peptides and induces resistance pathways through the transcription factor σ_E_ and the activator DegS [277]. Furthermore, studies have demonstrated that OmpA confers resistance to polymyxin B and protamine in *K. pneumoniae* [212]. The only case where porins have been directly involved in resistance to AMPs is observed with *E. coli,* where blocking OmpF by the simultaneous addition of spermine or cefepime inhibits colicin action [187]. Interestingly, the presence of a gap formed by OmpF facilitates the insertion of AMPs into the LPS, enabling them to establish hydrogen bonds with the phosphate groups of the inner core oligosaccharides. OmpF was found to play a crucial role in the entry of AMPs such as P6, either by elaborating the binding site for LPS or by directly transporting AMPs across the outer membrane [188] (Table 2). Moreover, Zhang et al. discovered that the downregulation of OmpW mediated by SoxS, a transcriptional factor involved in oxidative stress, contributes to resistance against oxidative stress and may also play a role in resistance against AMPs in *E. coli* [474]. A reduction in the expression of OmpW was also observed in a colistin-resistant mutant of *A. baumanni* [475]. Finally, OmpA-like proteins were show to confer AMP resistance in many other Gram-negative pathogens (Table 2).

#### 3.4.3. Peptidoglycan Modifications and AMP Resistance

To reach their target structures inside bacterial cells, particularly in Gram-positive bacteria, AMPs must penetrate through the dense layer of peptidoglycan. The physicochemical properties and density of this layer are pivotal in determining bacterial sensitivity to antimicrobial agents [476].

In *Salmonella* and *E. coli,* the N-acetylmuramoyl-l-alanine amidases, encoded by *amiA* and *amiC*, contribute to increased resistance to protamine, magainin 2, and melittin. The expression of these genes is governed by the twin arginine translocation (Tat) system. Conversely, the CpxR/CpxA two-component system is regulated by the overexpression of the *nlpE* gene, which encodes an outer membrane lipoprotein involved in copper homeostasis and adhesion. This system upregulates *amiA* and *amiC* expression, thereby enhancing resistance [189].

The N-deacetylation of peptidoglycan by the peptidoglycan N-deacetylase (encoded by the *pgdA* gene) in *L. monocytogenes* is a crucial mechanism for evading the host innate immune response. Boneca et al. (2007) demonstrated that mutants of *L. monocytogenes* lacking PgdA are highly susceptible to lysozyme activity, rapidly eliminated by macrophages, and elicit a significant IFN-β response through the TLR2 and Nod1 pathways [329]. In *S. aureus*, the carbon at position 6 of the N-acetylmuramic acid is acetylated by the O-acetyltransferase Oat, which grants these bacteria resistance to the muramidase activity of lysozyme [352]. In group B streptococcus (GBS), a mutant of the *ponA* gene, encoding for the penicillin-binding protein 1a (PBP1a) (both transglycosylase and transpeptidase), exhibits high sensitivity to defensins and LL-37 [323]. Furthermore, a mutation in the *pgm* gene, which encodes a phosphoglucomutase essential for preserving peptidoglycan integrity in *Streptococcus iniae*, increases the bacterial susceptibility to AMPs [380]. N- and O-deacetylations of peptidoglycan have been linked to AMP resistance in many other bacterial pathogens (Table 1 and Table 2).

#### 3.4.4. Modifications of Teichoic Acids and AMP Resistance

TAs constitute the second major component of the cell wall in Gram-positive bacteria, comprising more than 50% of the cell wall mass. TAs are polyanionic polymers that exist in many forms, such as wall teichoic acids (WTAs), which are covalently bound to the peptidoglycan and are presented on the outer surface of bacteria, and lipoteichoic acids (LTAs), which are anchored to glycolipids of the cytoplasmic membrane [477,478]. Mutants of *S. aureus* deficient in WTA exhibits up to a 100-fold increase in resistance to degradation and killing by gIIA phospholipase A2 (PLA2) and human β-defensin 3 (HBD-3) compared to the WT (Table 3). However, these mutants maintain sensitivity similar to the WT to other cationic AMPs, including Magainin 2 amide, HNP1-3, LL-37, and lactoferrin [358].

Specific enzymes called WTA glycosyltransferases mediate the glycosylation of WTA in *L. monocytogenes*. These enzymes catalyze the transfer of sugar molecules, such as rhamnose and N-acetylglucosamine in serovar 1/2a, and galactose and glucose in serovar 4b, into the WTAs, thereby modifying their structure and leading to resistance to AMPs [331,332]. On the other hand, many studies have revealed that the D-alanylation of TAs reduces the overall negative charge of the bacterial surface and confers resistance to AMPs (Figure 4) [479] (Table 3). This substitution is also crucial for stimulating TLR2 [480,481], and the absence of such a substituent leads to a significant decrease in bacterial inflammatory properties [482]. The incorporation of positively charged D-alanine residues into LTAs is catalyzed by products of the *dlt* operon [479]. This operon has been characterized in at least thirteen species of Gram-positive bacteria where it confers resistance to various AMPs (Table 3). The *dlt* operon generally includes the genes *dltA*, *dltB*, *dltC,* and *dltD*, all of which are essential for the D-alanyl esterification of TAs (Figure 4). The D-alannylation of TAs is completed in two steps: first by the D-Alanine-D-alanyl carrier protein ligase (Dcl), encoded by the *dltA* gene, and then by the D-alanyl carrier protein (Dcp), encoded by the *dltC* gene. The *dltB* and *dltC* genes are also indispensable in this D-alanylation process. Depending on the bacterial species, the content of D-alanyl ester in WTAs and LTAs depends, among other factors, on the pH of the environment, temperature, and concentration of NaCl and KCl salts [479].

The inactivation of the *dltA*, *dltB*, *dltC*, or *dltD* genes in the non-pathogenic bacterium *B. subtilis* results in mutants with WTAs and LTAs devoid of D-alanyl ester residues [295]. The knock-out mutations of the *dlt* operon in many Gram-positive species have been shown to significantly reduce resistance to both natural and synthetic cationic AMPs (Table 3). Furthermore, many *dlt* mutants are severely impaired in their virulence in animal models and their ability to adhere to and invade several eukaryotic cell lines in vitro, probably due to the highly electronegative surface of the bacteria and/or altered adhesin-binding activities [301,333] (Table 3). These features also suggest that D-alanine esterification of TAs contributes in various ways to the ability of some bacteria to circumvent mucosal and systemic antimicrobial defenses to produce systemic infections. Although the pathogen *S. pneumoniae* R6 contains phosphorylcholine esters instead of D-alanyl esters in its TAs [483], the *dlt* operon has been identified in its genome and confers resistance to cationic AMPs [484]. The inactivation of *dltA* in these pneumococci increases bacterial sensitivity to antibacterial molecules (chromatin and protein granules) from neutrophil extracellular traps (NETs) [485]. The reduction in D-alanyl esters in teichoic acids within *Lactobacillus reuteri* was also shown to decrease resistance to nisin and to impair the colonization of the mouse gastrointestinal tract [486].

The control of *dlt* gene expression varies among different Gram-positive bacterial species because they colonize different niches and adapt to different growth and stress conditions. For example, the *dlt* operon of *B. subtilis* is part of the σ_x_ regulon, and its expression also depends on the global regulators SpoOA and AbrB [294,295]. Additionally, Poyart et al. (2001) showed that the activation of the DltSR TCS depends on the amount of D-alanine available in the cytoplasm, providing resistance to cationic peptides by increasing cell wall density [327,328]. In *S. aureus*, *dlt* operon transcription is repressed in response to high concentrations of Na^+^ as well as moderate concentrations of Mg^2+^ and Ca^2+^ in the extracellular environment, while the *dltD* gene is derepressed (58-fold) by the *agr* locus (accessory gene regulator) encoding the Agr quorum sensing regulator [366]. The GraRS TCS has also been shown to control D-alanylation in *S. aureus*, conferring resistance to host AMPs [367]. The regulation of the *dlt* operon in *S. epidermidis* and *S. aureus* is also under the control of a three-component regulation system, ApsSX/ApsR (Aps, antimicrobial peptide sensor), homologous to GraRS. The sensor AspX is highly specific in recognizing and binding cationic AMPs, which subsequently induces the expression of other genes involved in AMP resistance, including *mprF* and the ABC-type VraFG transporters [368,369] (Figure 4). The regulation of the *dlt* operon in *L. monocytogenes* is controlled by the regulator VirR belonging to the pleiotropic VirRS regulation system [334]. Finally, *S. pneumoniae* upregulates the *dlt* locus through the CiaRH sensoring system, which senses stresses caused by AMPs, resulting in the D-alanylation of TAs and increased inflammatory responses [387].

### 3.5. Role of the Cytoplasmic Membrane Phospholipids and Their Modifications in Resistance to AMPs

The primary lipids found in the cytoplasmic membrane of bacteria consist of anionic PG, CL (also known as diphosphatidylglycerol, synthesized by cardiolipin synthase), and the neutral or zwitterionic PE. For example, the membrane lipid composition of *E. coli* comprises predominantly 80% PE and only 15% PG, in contrast to *S. aureus*, which lacks PE and contains 58% PG and 42% CL [487]. These variations in phospholipid content may account for the ineffectiveness of some cationic antimicrobials against bacteria. In particular, the alteration of CL levels within membranes has been proposed as a mechanism through which pathogens develop resistance to cationic AMPs [488]. Molecular dynamics simulations of the interaction between the short AMP aurein 1.2 and an anionic CL-containing lipid bilayer showed that the structural properties of CL, including rigidity, exposed charged phosphate groups, and the promotion of negative membrane curvature, oppose aurein 1.2’s membrane destabilization mechanism, potentially contributing to bacterial resistance against positive membrane curvature-dependent AMPs [12]. In fact, negative curvature-inducing lipids, like CL and phosphatidylserine, block the lytic activity of short AMPs, like aurein 1.2, magainin 2, polybia-MP1, LL-37, and ΔM2. This hindrance requires higher peptide-to-lipid ratios for peptide-induced transmembrane pore formation compared to PG [12]. Furthermore, a minor amount of phosphatidic acid (anionic), lysyl-phosphatidylglycerol (LPG), which bears a positive charge, and glycolipids are also present in bacterial membranes [489]. Other mechanisms within the cytoplasmic membrane, such as alterations in lipid charge and membrane energetics, also contribute to bacterial resistance to AMPs.

The first observations regarding modifications of bacterial cytoplasmic membrane phospholipids in response to environmental cues were made in *P. fluorescens*. It has been shown that *P. fluorescens* can adapt to cationic AMPs by altering its cytoplasmic membrane. Transitioning *P. fluorescens* from a phosphate-rich to a phosphate-deficient environment led to a significant reduction in the levels of PE, PG, and CL in the cytoplasmic membrane. This shift in membrane composition, particularly in anionic phospholipids, was followed by the emergence of a cationic lipid component containing ornithine. The resistance of *P. fluorescens* to polymyxin B was found to be proportional to the amount of this lipid found in the cytoplasmic membrane of these bacteria [259]. Cao and Helmann (2004) identified the *pss-ybfM-psd* operon as part of the σ_X_ regulon in *B. subtilis*. This operon is involved in PE biosynthesis, which, when incorporated into the bacterial cytoplasmic membrane, decreases its overall net negative charge, leading to a reduced affinity for cationic AMPs [294]. Other Gram-positive bacteria, such as certain *Staphylococcus* species, consistently express membrane phospholipids with reduced negative charge [490]. The analysis of the constitutive phospholipid composition in several of these species reveals predominantly polar lipid profiles comprising PG and CL. Among the staphylococci tested, *S. aureus* stands out for its lipid composition enriched in unsaturated menaquinones with eight isoprene units, and LPG, a derivative of PG that exhibits considerably lower electronegativity [491]. In staphylococci, resistance to daptomycin by the alteration of membrane phospholipid composition is postulated to change the fluidity of the membrane, thus interfering with daptomycin binding and subsequent oligomerization. Indeed, by analyzing the action of a membrane active AMP on giant unilamellar vesicles (GUVs), increasing LPG levels did not correlate with reduced peptide binding by electrostatic repulsion, but with the inhibition of intravesicular dye leakage post-binding, indicating a protective effect on membrane integrity [492]. Considering the protective role of phospholipids and fatty acid types in regulating membrane fluidity, it is plausible that CL contributes to preventing daptomycin translocation upon insertion into the membrane [493]. Mutations that modify enzyme function could potentially influence daptomycin resistance by shifting the PG to CL ratio in the cell membrane. Supporting this notion, the genomic analysis of 33 daptomycin-resistant *Staphylococcus* strains revealed associations between mutations in genes such as *pgsA* (involved in PG synthesis) and *cls2* (cardiolipin synthase) and daptomycin resistance [370] (Figure 5G) (Table 3). Moreover, Bayer et al. (2000) demonstrated that *S. aureus* strains resistant to tPMP-1 (thrombin-induced platelet microbicidal protein 1) in vitro exhibit a notable increase in unsaturated membrane lipids compared to genetically similar strains that are more susceptible to this cationic AMP [494].

Several Gram-positive bacteria mitigate the negative charge of PG by replacing it with a cationic residue, lysine, forming LPG [371] (Figure 5G) (Table 3). The enzyme responsible for this incorporation is the product of the *mprF* (multiple peptide resistance factor) gene, known as the LPG synthase MprF. MprF is a bifunctional enzyme, consisting of a carboxy-terminal cytoplasmic tail responsible for the lysinylation of PG using lysyl-tRNA as a donor and an amino-terminal domain that facilitates “flippase” activity, transporting LPG from the inner to the outer membrane. Additionally, a central domain appears to assist in both lysinylation and flippase activities [495].

*S. aureus* responds to daptomycin by increasing its overall cell-surface charge, likely to repel daptomycin insertion and maintain membrane integrity, a process facilitated by mutations in *mprF*. These mutations, resulting in amino acid changes clustering in the central bifunctional region that overall confer a “gain-of-function” to the enzyme [496], lead to a gain-of-function phenotype, enhancing the synthesis of positively charged LPG. Studies have also demonstrate that expressing *mprF* with daptomycin-resistant mutations (but not WT *mprF*) restores daptomycin resistance in *mprF* mutant strains of *S. aureus* [497]. Additionally, inhibiting MprF protein synthesis by antisense RNA (directed against *mprF* transcripts) reverses daptomycin resistance in vitro in strains with gain-of-function mutations [498].

On the other hand, the exposure of *S. aureus* to subinhibitory levels of magainin 2 and gramicidin D prompts the bacterium to develop resistance against these peptides in a MprF-dependent manner [372]. The inactivation of the *mprF* gene in *S. aureus* results in the depletion of membrane LPG, heightening the bacterial susceptibility to defensins and PLA2 by accumulating significantly more surface-bound cationic AMPs than WT bacteria [371,499,500]. Similarly, human neutrophils containing a high quantity of α-defensins deactivate Δ*mprF* mutants of *S. aureus* much more rapidly and effectively than WT bacteria [371] (Table 3).

Homologues of MprF have also been identified and functionally characterized in *B. anthracis*, *L. monocytogenes*, and *Enterococcus* sp. [290,335] (Table 3). The lysylation of PGs in *L. monocytogenes* depends on the VirR response regulator, which is critical for the virulence of this bacterium [334]. Furthermore, the genomes of *E. faecalis* and *E. faecium* encode two paralogs, MprF1 and MprF2, where MprF2 seems to play a primary role in PG aminoacylation in *E. faecalis*. The absence of L-PG from *E. faecalis* membrane was shown to increase bacterial susceptibility to cationic AMPs [302,303,501] (Table 3). These findings propose a model where the targeted action of AMPs correlates with their effectiveness in killing bacteria and interfering with the assembly of virulence factors. Moreover, Reyes et al. (2015) demonstrated that the deletion of the gene encoding the response regulator LiaR, a component of the LiaFSR system controlling cell envelope homeostasis, from daptomycin-resistant *E. faecalis*, altered the CL microdomain localization in the cell membrane. This deletion led to the hypersusceptibility to daptomycin and telavancin as well as to various AMPs of diverse origins and mechanisms of action. The observed changes in susceptibility to these AMPs were associated with reduced virulence in a *Caenorhabditis elegans* model [305]. Recent in vitro evolution experiments targeting daptomycin resistance unveiled numerous novel mutations associated with resistance, notably including mutations in the protease-encoding *ftsH* gene, which were found to be enriched exclusively in a Δ*mprF1* Δ*mprF2* background (slowed evolution to daptomycin resistance). Moreover, it was shown that FtsH indirectly modulates the levels of the chaperone operon repressor (HrcA), consequently affecting the pace of daptomycin resistance evolution [304]. Interestingly, Klein et al. (2008) demonstrated that membrane PGs from both the outer and inner membranes of *P. aeruginosa*, when cultured in acidic conditions, undergo substitution with alanine to form alanyl-phosphatidylglycerols (APGs). This substitution is catalyzed by an APG synthase encoded by the PA0920 locus identified in the genome of *P. aeruginosa*, which utilizes alanine tRNAs as alanine donors. The addition of alanine to PG reduces membrane permeability and neutralizes the negative charge of PG, consequently reducing its affinity for protamine [252,253]. Recently, an orthologue of *S. aureus* MprF, known as LysX, was identified in *Mycobacterium tuberculosis*, suggesting its potential membership in the aminoacyl-phosphatidylglycerol synthase family. LysX plays a critical role in Lys-PG formation, conferring resistance against cationic AMPs and a low pH. The deletion of *lysX* leads to disruptions in membrane potential, growth impairment, and defects in intracellular replication [500]. Additionally, another LysX orthologue, named LysX2, is exclusive to pathogenic mycobacteria and has been demonstrated to decrease the surface negative charge under acidic conditions, enhance bacterial cell viability at lethal pH levels, and hinder biofilm formation [502].

### 3.6. Active Efflux and Transport of AMPs

When the previously outlined mechanisms fail and AMPs amass on bacterial membranes, they might undergo conformational changes upon interacting with phospholipids. Upon reaching a certain threshold, they could breach the periplasm or cytoplasm via transient pore formation [503]. Even in such scenarios, bacteria retain a last resort mechanism to eliminate AMPs, orchestrated by efflux pumps and transporters. This includes the ability to either extrude AMPs that have crossed the outer membrane intact to prevent their intracellular accumulation [156,223,266,268,283,504,505,506,507,508], or transport them intracellularly for degradation by cytoplasmic proteases, such as those facilitated by the Sap (Sensitive to Antimicrobial Peptides) transporter (for a detailed review, see [471]). Efflux pumps are active transporters able to recognize and expel a wide array of structurally diverse compounds, depending on their substrate specificity conferred by the presence of one or multiple drug-binding sites [509]. These pumps not only play a role in resistance against toxic agents, but also in regulating physiological functions and can contribute to the formation of biofilm and virulence [510]. Efflux pumps are classified into seven widely acknowledged categories: (i) ATP-binding cassette (ABC) superfamily; (ii) major facilitator system (MFS) superfamily; (iii) multidrug and toxic-compound extrusion (MATE) family; (iv) drug/metabolite transporter superfamily (DMT), which encompasses the small multidrug resistance (SMR) family; (v) resistance–nodulation–division (RND) superfamily; (vi) proteobacterial antimicrobial compound efflux (PACE) family; and (vii) the p-Aminobenzoyl-glutamate transporter (AbgT) family [511]. Many studies highlighted the implication of ABC-type transporters and RND-type efflux pumps in bacterial resistance to AMPs, while only few others reported the contribution of MFS family members to this phenotype [510] (Table 2 and Table 3).

Bacterial ABC transporters are categorized into exporters and importers [512]. Among them, those responsible in transporting AMPs display diverse structural configurations, such as the tripartite structures found in Gram-negative bacteria, as seen in the MacAB–TolC efflux pump [513]. Peptide substrates recognized by MacB and its homologues typically resemble small disulfide-bonded peptides, reminiscent of certain AMPs like mammalian defensins. The expression of MacB-like proteins has been shown to impact the survival of *Salmonella* and *S. pyogenes* within macrophages [265,319] (Table 2 and Table 3). Notably, the transcription of *macAB* is upregulated during macrophage infection and following exposure to AMPs, a response supported by PhoPQ. The constitutive expression of *macAB* enhances the survival of *Salmonella* in the presence of the AMP C18G [266]. Moreover, the multifunctional inner membrane protein complex Sap transporter serves the purpose of importing various AMPs into the cytoplasm of various Gram-negative bacterial pathogens (Table 2). It consists of SapA, which functions as a periplasmic solute-binding protein, along with SapB and SapC, acting as transmembrane proteins to create a pore in the inner membrane. Additionally, SapD and SapF serve as ATPase subunits, while SapA is an integral membrane protein likely associated with SapC [267]. SapA binds directly to AMP and transports it from the periplasm to the SapBCDF complex for further transfer into the bacterial cytoplasm, where the AMPs undergo degradation and their amino acids are recycled [196,197,200,213,267,514,515,516].

In Gram-positive bacteria, particularly *S. aureus*, the GraSR TCS controls the expression of the adjacent ABC transporter, *vraFG*, which, while not conferring resistance independently, plays a vital role in sensing cationic AMPs and activating the GraR-dependent transcription of *dlt* and *mprF* genes [376,517] (Table 3).

The efflux pumps of the RND superfamily, renowned for their polyselective nature, have emerged as clinically significant determinants, conferring MDR in numerous bacterial pathogens [509]. RND pumps, powered by the proton motive force (PMF) and reliant on the pH gradient across the inner membrane, form a tripartite structure with periplasmic adaptor proteins and outer membrane protein channels, such as TolC, to facilitate the export of substrates from the cell [509,511,518]. The most studied RND systems involved in AMP resistance include the AcrAB–TolC of Enterobacterales species, Mex of *P. aeruginosa*, Vex of *Vibrio*, and Ade of *Acinetobacter* (Table 2). Moreover, there is increasing evidence supporting the capability of the MtrCDE pump to recognize and extrude AMPs in various bacteria, including *N. gonorrhoeae*, *N. meningitidis*, and *H. ducreyi* [197,221,222,223,224,229] (Table 2). MtrD appears to be the only RND pump protein for which mutagenesis studies have identified key residues crucial for the binding of an AMP, polymyxin B [222,519], suggesting that the latter is an actively extruded substrate of this pump.

Despite its broad specificity, the involvement of AcrAB–TolC in the AMP resistance of *E. coli* has been a contentious subject, as evidenced by various studies. For instance, a study performed in 2009 concluded that LL-37, polymyxin B, and various defensins were not substrates of AcrAB–TolC [520]. However, a subsequent study in 2010 suggested otherwise, indicating increased susceptibility to these AMPs in *acrAB*-deficient mutants [521]. Warner and Levy (2010) revealed that the discrepancies in AMP susceptibility among WT and the *acrAB* and *tolC* mutants noted by Rieg and Warner were attributed to variations in microbiological media [521], with differing ion concentrations potentially affecting AMP stability and binding to negatively charged surfaces [522,523]. Moreover, the increased susceptibility of *tolC*-deficient mutants implied the participation of other efflux pumps utilizing TolC as a mediator for AMP efflux. Additionally, the CpxR/CpxA TCS has been demonstrated to upregulate the multidrug resistance cascade, enhancing *E. coli* resistance to a model AMP [191]. Despite these findings, there remains a gap in the literature regarding specific studies identifying AMP-binding sites within the AcrAB efflux pump in Gram-negative bacteria, unlike the situation with the MtrCDE pump. The majority of research on AcrAB centers on its interactions with antibiotics and biocides rather than AMPs [511,518,524,525]. Considering the evidence demonstrating that AcrAB–TolC can expel large molecules, such as macrolide antibiotics (with a molecular weight smaller than 1000 Da) [526,527], it is plausible that cyclic AMPs like colistin and polymyxin B, sharing a similar size (approximately MW 1200 Da), could also be substrates of this pump. However, this hypothesis does not apply to larger AMPs such as LL-37 (4493 Da) or defensins (3000–5000 Da) [471]. Numerous studies utilizing isogenic *acrAB* mutants (lacking AcrAB proteins), loss of efflux function mutations (possessing non-functional AcrAB proteins), and efflux pump inhibitors have shown that AcrAB contributes to polymyxin resistance in many Gram-negative pathogens (Table 2). Yet, it remains unclear whether this contribution arises from a direct efflux mechanism of the AMP itself, or indirectly through the transport of other substrates affecting the bacterial surface charge, or alterations in membrane permeability beyond simply the loss of efflux, which may also affect susceptibility [521,528]. This uncertainty persists due to the physiological multifunctionality of AcrAB and its dependence on stress-induced regulators, like CpxR, Mar, Ram, Rob, and Sox [509,518]. For instance, an *acrB* knockout mutant of *K. pneumoniae* displayed a significantly increased susceptibility to AMPs [214], as well as a reduced ability to cause pneumonia in a mouse model, compared to the WT strain [214] (Table 2). In highly colistin-resistant clinical strains of *K. pneumoniae*, a newly discovered unusual RND pump (H239_3064), which lacks a known periplasmic adaptive protein but shares 49% amino acid identity with the parental AcrB, has been associated with colistin resistance. This pump is highly induced by the CrrAB TCS, which harbors missense amino acid substitutions in the CrrB histidine kinase, leading to a reduced susceptibility to colistin. The deletion of H239_3064 in the crrB background resulted in an 8-fold decrease in colistin MIC [215] (Table 2).

In addition to *Klebsiella*, other members of the ESKAPE pathogens, such as *Enterobacter asburiae* and *Enterobacter cloacae*, partly depend on the AcrAB-TolC pump to exhibit polymyxin heteroresistance [168]. An AcrB ortholog, KexD, similar to H239_3064 of *K. pneumoniae*, was discovered in various *Enterobacter* species, including *E. bugandensis*, and linked to colistin resistance [529] (Table 2). KexD interacts with the predicted small transmembrane protein CrrC via its membrane domain. Comparable interactions were simulated for AcrB and AcrD efflux pumps. Recently, García-Romero et al. (2024) proposed a model in which drug efflux, enhanced by CrrC interactions with major efflux pumps, along with lipid A modifications, regulated by PhoPQ and CrrAB, confer high-level resistance and heteroresistance to polymyxin B in *E. bugandensis*. They demonstrated that KexD and CrrC play a significant role in polymyxin B resistance and heteroresistance, with their genes being highly overexpressed in response to this AMP. Through co-immunoprecipitation experiments and proteomic analysis, they suggested that TolC and AcrA interact with KexD, indicating that KexD likely functions with AcrA and TolC. This conclusion is supported by the increased polymyxin B susceptibility of the *acrAB* and *tolC* mutants. Additionally, the increased susceptibility to polymyxin B of *crrCkexD* and *crrC* mutants does not depend on L-Ara4N modifications. Moreover, the stronger effect of *crrCkexD* on reducing polymyxin B resistance suggests that both proteins have a synergistic effect [530]. Colistin heteroresistance in a clinical isolate of *A. baumannii* was also associated with the overexpression of another RND component, AdeB, highlighting the involvement of the AdeABC system when exposed to colistin [147] (Table 2).

In *P. aeruginosa*, although MexA shares a high similarity with AcrA, and MexB with AcrB (71% and 89%, respectively), Pamp et al. (2008) reported that the adaptation of *P. aeruginosa* to colistin in vitro depends on the MexAB-OprM efflux pump only in bacteria growing as biofilms and has not been observed in the same bacteria maintained in a planktonic state. Specifically, the induction of the MexAB-OprM pump was observed only within the metabolically active bacterial subpopulation located on the surface of biofilms [254]. However, other efflux pumps in *P. aeruginosa*, such as MexCD–OprJ and MexXY–OprM, were found ineffective in expelling polymyxin B [255]. In *C. jejuni*, a mutation in the inner membrane component-encoding *cmeE* gene of the CmeDEF efflux pump led to increased sensitivity to polymyxin B. This sensitivity was observed consistently in the *cmeF/cmeB* double mutants, which lack the inner membrane component of the primary efflux pump CmeABC [165] (Table 2). Furthermore, a single mutant affecting *acrA* and a double mutant affecting both *acrA* and the membrane fusion protein-encoding *mdtA* gene of the MdtABC RND efflux pump in the insect pathogen *Photorhabdus laumondii* exhibited at least an 8- to 16-fold decrease in colistin and polymyxin B MICs, while resistance to cecropins A and C was not affected compared to the parental strain. The authors suggested that AcrAB indirectly contributes to AMP tolerance at supra-physiological concentrations, likely by maintaining membrane integrity rather than through direct efflux, which is probably irrelevant for *Photorhabdus* ability to resist AMP-induced killing in the insect hemolymph [231,232] (Table 2).

The efflux of polymyxin B, cecropin P1, and melittinin in *Yersinia enterocolitica* seems to occur through the RosA MFS efflux pump coupled to the RosB potassium antiporter, involving TolC. The RosA/RosB regulon appears to be inducible by cationic AMPs and temperature (37 °C), enabling bacterial adaptation to an acidic environment rich in cationic AMPs, such as the phagolysosome [282]. Furthermore, the MFS-type *emrB* knockout mutation in *A. baumannii* resulted in an increased susceptibility to colistin compared to the WT strain [146]. Also, the EmrAB–TolC system confers resistance to protamine in *E. coli*, as demonstrated by the notable difference in survival rates between the *emrB* (20%) and *tolC* (0%) mutants when exposed to protamine [191]. The EmrAB/TolC and AcrAB/TolC efflux systems simultaneously contribute to *E. coli* protamine resistance in a CpxR/CpxA-dependent manner through the activation of *mar* operon transcription. Moreover, CpxR/CpxA induces the transcription of the *aroK* gene, enhancing the production of aromatic metabolites that release the MarR repressor from the *marO* site, thereby increasing *marA* expression and subsequently triggering the efflux pump-mediated multidrug resistance cascade [191].

MFS efflux systems have also been associated with cationic AMP resistance in Gram-positive bacteria, such as staphylococci [531]. The pSK1plasmid confers the resistance of staphylococci to numerous organic cations through an MFS pump encoded by a plasmid gene. Kupferwasser et al. (1999) showed that transferring the *qacA* gene from *S. aureus* to another more sensitive parental strain increases its resistance to tPMP-1 (but not to α-defensins and protamine). Additionally, the mere presence of the *qacA* gene product seems sufficient to confer this resistance, independently of the efflux mechanism itself, especially as tPMPs do not appear to be the major substrate of this pump [375]. This possibility suggests that the QacA protein could modify the composition of the cytoplasmic membrane to avoid disruption by tPMP-1.

Another system relevant to AMP resistance is the ubiquitous TrkG/H–TrkA potassium uptake system, functioning through K^+^ uptake coupled with H^+^ symport [532]. Notably, a *trkA* isogenic mutant of the highly virulent marine bacterium *Vibrio vulnificus* displayed increased susceptibility to protamine and polymyxin B compared to the WT. Furthermore, infection trials revealed reduced virulence in both normal and iron-treated mice following the intraperitoneal or subcutaneous administration of this mutant [279] (Figure 5A).

### 3.7. Alteration of Membrane Energetics and Resistance to AMPs

The activities of several types of AMPs are affected by the transmembrane potential [533]. Some microbial pathogens utilize the regulation of their energy status by altering membrane energetics as a strategy to evade the action of AMPs. For instance, type II defensins show significantly lower activity against organisms with an inherently reduced transmembrane potential Δψ (due to respiration deficiency), or those capable of adapting to such conditions. Therefore, *S. aureus* strains with constitutively reduced Δψ demonstrate decreased sensitivity to specific AMPs [534]. Furthermore, cytochrome c oxidase modulates the transmembrane potential in *C. jejuni*, affecting membrane permeability to polymyxin B [164]. Disulfide reductase has also been implicated in AMP resistance in *S. enterica* [535].

### 3.8. Cellular Differentiations and Resistance to AMPs

Some bacteria exhibit susceptibility to AMPs in one morpho-physiological state while demonstrating resistance in another. This difference in susceptibility is attributed to variations in behavior between the planktonic and aggregated forms of bacteria. Notably, the development of small colony variants (SCVs) and the formation of biofilms play significant roles in this resistance mechanism [254,536]. These phenomena, observed in various pathogenic bacteria, directly or indirectly contribute to their ability to resist the antimicrobial effects of AMPs, posing challenges for effective treatment strategies.

#### 3.8.1. Small Colony Variants and Niche-Specific Resistance to AMPs

Small colony variants (SCVs) are a subset of bacteria characterized by slow growth and distinct phenotypic and pathogenic features under environmental stress. They exhibit unique colony morphology and biochemical traits, making their identification challenging. Clinically, SCVs demonstrate reduced susceptibility to antimicrobial agents compared to typical bacterial strains and may contribute to latent or recurrent infections [536]. Numerous studies have delved into the resistance of *S. aureus* SCVs against AMPs. The SCV phenotype in *S. aureus* can be triggered within the intracellular microenvironment at a frequency of 10^−3^, significantly higher than the spontaneous rate of less than 10^−7^, aiding in the evasion of the host immune response [537]. There is evidence indicating that reductions in cellular energetics detrimentally affect the effectiveness of AMPs relying on Δψ for their mechanism of action or target affinity. Proctor et al. (1998) illustrated how *S. aureus* reversibly adopts this advantageous strategy to survive within the microenvironment of vascular endothelial cells by forming SCV colonies [537,538,539]. These variants typically exhibit atypical morphology, electron transport defects, and diminished envelope affinity for cationic antimicrobial agents. Mutants of *S. aureus* strains with reduced Δψ display significantly higher resistance to AMPs compared to their parental counterparts [538,539]. Furthermore, clinical *S. aureus* strains sensitive to tPMP1 exhibit slower multiplication rates in cardiac tissues and splenic abscesses, whereas no discrepancy in multiplication is observed at the renal level. This discrepancy is attributed to the relatively diminished protective role of tPMP1 in environments with high osmotic forces, such as the kidneys [35]. A similar mechanism is observed in *P. aeruginosa*, frequently infecting tissues where salt transport dysfunctions elevate the local ion content, particularly in the respiratory tract of cystic fibrosis patients, where airway fluids contain abundant salts and AMPs [35]. Such observations underscore the risk of certain pathogens exploiting specific tissues or physiological microenvironments to evade the action of AMPs.

Additionally, Samuelsen et al. (2005) demonstrated that *S. aureus* SCVs exhibit acquired resistance to lactoferricin B, primarily due to metabolic variations. Hemin, menadione, or thymidine SCV *hemB* auxotrophic mutants of *S. aureus* have been found to be more susceptible to AMPs compared to bacteria having one of these components [540]. Furthermore, Glaser et al. (2014) have shown that clinically derived *S. aureus* SCVs as well as a *hemB* auxotroph *S. aureus* SCVs are less susceptible to various human skin-derived AMPs, such as human β-defensin and LL-37 [541].

#### 3.8.2. Biofilm Formation and Resistance to AMPs

Some AMPs, such as the human peptide LL-37 and a novel synthetic cationic peptide named 1037, comprising just nine amino acids, can hinder biofilm formation even at concentrations lower than their MIC. The 1037 peptide effectively hindered biofilm formation by more than 50%, notably against the Gram-negative pathogens *P. aeruginosa* and *B. cenocepacia*, as well as the Gram-positive bacterium *L. monocytogenes* [542]. However, bacterial implantation into biofilms triggers changes in gene expression, resulting, among other effects, in a greater resistance to antimicrobial agents compared to individual bacteria. Mechanisms behind biofilm-mediated AMR resistance involve multiple factors simultaneously, such as shielding by the polysaccharidic extracellular polymeric substance (EPS), nutrient deficiency and altered environment, the formation of persister cells, extracellular DNA release, stress response, and enhanced efflux pump activity [543,544,545,546]. The biofilm environment also increases the mutation rate and exchange of genetic material, enabling the transfer of resistance traits on mobile genetic elements [547,548].

*P. aeruginosa* produces significant amounts of a highly anionic exopolysaccharide known as alginate. Friedrich et al. (1999) purified alginate and demonstrated its interference with the activity of cationic AMPs [549] (Table 2). Alginic acid entraps bacterial cells, contributing to the formation of biofilms involved in the development and persistence of several chronic bacterial infections in animals and humans, particularly in the lungs during cystic fibrosis [550,551]. The fact that the activity of several cationic AMPs is inhibited in the presence of mono- and divalent cations could enhance the protective role of alginate [35]. However, *S. epidermidis* produces an atypical anionic extracellular polysaccharide called PIA (polysaccharide intercellular adhesin), which repels AMPs secreted onto the skin (except for the anionic dermcidin) in the presence of low saline concentrations (Table 3). However, it enables resistance to dermcidin only in salt-rich microenvironments, such as the skin [378]. A study performed by Begun et al. (2007) examined the role of PIA in protecting against *C. elegans* immune system and the production of lethal infections [552]. Induced resistance factors in biofilms include those resulting from induction by the antimicrobial agent itself [553]. The slow growth (quiescence or dormancy) of the bacteria inside biofilms, particularly in the deeper layers, likely stems from nutrient and oxygen deficiency, leading to an extremely reduced metabolic state. This reduced metabolic activity renders the bacteria less receptive to antimicrobial agents compared to metabolically active ones [554]. Efflux pumps are highly active in biofilms, essential for their formation and/or maintenance, and responsible for their multiple antibiotic tolerance/resistance [555,556]. Pamp et al. (2008) reported that the adaptation of *P. aeruginosa* biofilms to colistin in vitro occurs through the active expulsion of this peptide via the MexAB-OprM efflux pump. This mechanism is exclusively induced within biofilms and has not been observed in the same bacteria maintained in the planktonic state. Specifically, the induction of the MexAB-OprM pump has been observed only in the metabolically active bacterial subpopulation located on the biofilm surface [254]. Conversely, Folkesson et al. (2008) demonstrated that biofilm formation in *E. coli* confers a high tolerance to colistin on cells within the biofilm structure. However, this protection is conditional, depending on the structural and architectural organization of the biofilm, as well as the induction of specific tolerance mechanisms (not by colistin itself) involving the BasS/BasR regulatory system (*Salmonella pmrAB*) controlling the expression of the *ybf* operon (*Salmonella pmrHFIJKLM*) [557]. Furthermore, in the extracellular polysaccharide matrix of *P. aeruginosa* biofilms, much like in biofilms formed by other Gram-positive and Gram-negative bacteria, there is extracellular DNA (eDNA) among other components, likely released by bacteria or dead immune cells [558]. This eDNA (negatively charged) plays a role in shielding cationic AMPs [559]. When present at physiological concentrations, eDNA causes bacterial lysis by chelating the cations that stabilize bacterial LPS and cell membranes. Therefore, the genomic DNA released in sub-inhibitory quantities by lysed bacteria in the biofilm creates a cation-deficient environment. This deficiency leads to the induction of resistance to cationic AMPs (colistin and polymyxin B) via the activation of the PhoPQ and PmrAB TCSs [560].

Many studies have reported associations between biofilms and swarming motility [263,561]. Notably, the strong resistance to colistin and polymyxin B of swarming bacteria of *S.* Typhimurium has been linked to the induction under “swarming”-promoting conditions of the *pmrHFIJKLM* operon involved in polymyxin B resistance [562].

### 3.9. Manipulation of Host Cell AMP Production

In the absence of infections, the epithelia of higher animals and leukocytes produce small quantities of AMPs. However, this production is strongly induced in response to bacterial structures such as LPS and teichoic acids recognized by Toll-like receptors and may involve the NF-κB-dependent signaling pathway [563,564,565]. Indeed, it has been demonstrated that the LPSs of several bacteria, such as *E. coli*, *S*. Typhimurium, and *P. aeruginosa*, are capable of bypassing the induction of genes encoding AMPs [566]. Moreover, *S. pyogenes* is a very weak inducer of AMP production [320]. This observation suggests that *S. pyogenes* avoids recognition receptors or actively represses the induction pathway of β-defensin-2. An interesting study of the human gastrointestinal pathogen *Shigella dysenteriae* has revealed that the expression of LL-37 and β-defensin-1 is transcriptionally repressed in the early stages of infection. This plasmid-encoded repression was first observed in biopsies from patients with bacillary dysentery and then confirmed by experiments on cell cultures of monocytes and epithelial cells (Table 2). On the other hand, Arbibe et al. (2007) showed that virulent strains of *S. flexneri* repress the transcription of several genes encoding cationic AMPs of innate immunity (in particular, β-defensin and HBD-3, which are specifically active against *S. flexneri*) following the in vitro infection of polarized human intestinal cells [273]. This repression depends on the regulator MxiE of *S. flexneri* known to activate more than 10 genes encoding effectors of the type III secretion system (TTSS), including OpsF and OspG, which target signaling pathways via NF-κB and MAPK [273] (Table 2). The inhibition of cationic AMP production via MxiE has been confirmed in vivo at the transcriptional and translational levels in a murine model of human intestinal xenotransplantation [567]. Moreover, Krishnenduno et al. (2008) showed that the enteric pathogens *V. cholerae* and enterotoxigenic *E. coli* (ETEC) repress the expression of LL-37 and HβD-1 in intestinal epithelial cells while acting on signaling pathways known in eukaryotes. Indeed, cholera toxin (CT) and heat-labile toxin (LT), which are the two major virulence proteins of *V. cholerae* and ETEC, respectively, are primarily responsible for these repressions both in vitro and in vivo. CT represses the expression of AMPs at the transcriptional level by activating several intracellular signaling pathways involving protein kinase A (PKA), ERK MAPK, and Cox-2 [192] (Table 2).

Contrary to the inhibition of AMP production, another bacterial survival strategy may involve the stimulation of host mechanisms that counter-regulate the response by AMPs. Such a process has been demonstrated in the lungs of patients with cystic fibrosis, where the causative pathogen *P. aeruginosa* stimulates the accumulation of host cysteine proteases (cathepsins B, L, and S) in the respiratory tract fluids. These proteases degrade and inactivate human β-defensins 2 and 3, thereby promoting chronic infection and bacterial colonization characteristic of the disease [257].

*Ureaplasma* spp. are commonly found in the urogenital tract of adults and are associated with serious infections. Infections with *U. parvum* induce modifications in histone H3K9, particularly a decrease in histone acetylation. This alteration is linked to a downregulation of AMP gene expression, enabling bacteria to evade the host immune response, thereby leading to chronic infection [274] (Table 2). A similar mechanism involving histone modification, including the phosphorylation of H3 and deacetylation of H4, is observed in *L. monocytogenes*. This modification is induced by the *Listeria* toxin listeriolysin O in a pore-forming-independent manner. Comparable mechanisms are employed by *Clostridium perfringens* and *S. pneumoniae*, which utilize perfringolysin and pneumolysin, respectively [568]. Moreover, *Chlamydia* inhibits NF-κB activation by blocking the degradation of the NF-κB retention factor, IκBα, and preventing the nuclear translocation of NF-κB, ultimately repressing NF-κB transcription [569].

## 4. Conclusive Remarks and Future Directions

AMPs offer great potential in combating bacterial infections, yet they encounter significant challenges due to the easy development of resistance mechanisms, even to last-resort treatment options against MDR strains such as colistin. It is noteworthy that both Gram-negative and Gram-positive bacteria, whether pathogenic or commensals, have adopted various alterations or remodeling of their envelope structures to counteract AMP-mediated killing. As illustrated in this review, these cell surface modifications are evident across a wide range of bacterial species, including members of Enterobacterales, *P. aeruginosa, Neisseria* sp., *Bacillus* spp., *Lactobacillus* spp., *Streptococcus* spp., *Staphylococcus* spp., *L. monocytogenes*, and even *Mycobacterium* spp. These examples, along with other non-associated cell wall modifications, underscore the convergence of resistance mechanisms to AMPs by the common selective pressure exerted by these peptides. Therefore, modifications of bacterial cell surfaces appear to be the most conserved and effective resistance mechanism to AMPs. This is supported by numerous experimental observations indicating that spontaneous mutations and those introduced in structural and regulatory genes involved in these processes lead to the most in vitro AMP-hypersensitive phenotypes, along with the severe attenuation or abolition of virulence in various animal models. Understanding the intricate molecular pathways employed by bacteria to evade AMPs is paramount in developing effective strategies to combat resistance. Interdisciplinary approaches, such as genomics, metabolomics, structural biology, and bioinformatics, offer promising avenues to unravel the complexity of AMP resistance mechanisms.

From the limited research conducted to examine the speed of AMP resistance emergence and how resistance mechanisms would affect the fitness and virulence of mutants, it is well established that acquiring such resistance through mutation is not challenging for bacteria, with multiple facilitating mechanisms characterized to date. Some of these mechanisms have a minimal impact on bacterial fitness, thanks to compensatory mutations that can mitigate the associated fitness cost. These resistance mutations often confer cross-resistance to human AMPs, posing concerns about the therapeutic use of AMPs potentially selecting for mutants showing broad cross-resistance to diverse AMPs. This suggests that the routine clinical use of AMPs could select for resistant strains capable of evading the immune system. Researchers have emphasized the need for careful study and monitoring as AMPs are increasingly used in clinical settings. Therefore, thorough investigation and monitoring are essential to understand the implications of AMP resistance and to ensure their effective clinical use. Moving forward, novel therapeutic interventions should not only target bacterial resistance mechanisms but also strive to minimize the likelihood of resistance development through innovative peptide design and delivery strategies. Additionally, exploring alternative approaches, such as combination therapies with conventional antibiotics or AMP adjuvants, could enhance antimicrobial efficacy while mitigating the risk of resistance emergence.

Finally, fostering collaborative efforts across scientific disciplines is crucial to staying ahead of evolving resistance mechanisms and ensuring the continued efficacy of AMPs in combating bacterial infections. By embracing innovative future perspectives and leveraging synergistic approaches, we can enhance our arsenal against AMP resistance, ultimately advancing the fight against infectious diseases.

## Figures and Tables

**Table 1:** Selected antimicrobial peptides with bacterial membrane depolarization/disruption actions under clinical trials or approved for clinical application.

AMPs	Structure/Charge	Source	Amino Acid Sequence ^a^	Target Bacteria/Indications	Clinical Phase ^b^	Administration	Refs.
Polymyxin B	Cyclic/+5	*Bacillus polymyxa*	*6-mo-*DabTDabDab[γDablLDabDabT] (Polymyxin B1)	MDR G-infections	IV	Topical, oral, IV, ophthalmic, aerosolized	[101,102]
Colistin	Cyclic/+5	*Bacillus colistinus*	*6-mh-*DabTDab[γDablLDabDabT] (Colistin A)	MDR G-infections	IV	Topical, oral, IV	[103,104,105]
Nisin	Cyclic/+4	*Lactococcus lactis*	ITSISLCTPGCKTGALMGCNMKTATCHCSIHVSK (Nisin A)	Broad spectrum, food preservative	High clinical potential	Undefined; oral or IP in animal models	[106,107,108]
Daptomycin	Cyclic/-3	*Streptomyces roseosporus*	WNDTGKDADGSEY	G+ skin infections, endocarditis, & bacteremia	IV	IV	[109,110]
Gramicidin S	Cyclic/+2	*Brevibacillus brevis*	VKLFPVKLFP	Broad spectrum; wound infections, conjunctivitis, genital ulcers	In market	Ophthalmic and topical preparations	[111,112]
LTX-109 (Lytixar)	Cyclic/+3	Synthetic	R-Tbt-R-NH-EtPh	G+ skin infections, anti-MRSA and VRSA	II/III	Topical or nasal	[113,114]
Murepavadin (POL708)	Cyclic/+5	Synthetic	Ala-Ser-D-Pro-Pro-Thr-Trp-Ile-Dab-Orn-D-Dab-Dab-Trp-Dab-Dab	*Pseudomonas* in cystic fibrosis	III	IV and eFlow^®^ nebulizer system	[115,116]
LL-37 (hCAP18)	α-helical/+6	Human	LLGDFFRKSKEKIGKEFKRIVQRIKDFLRNLVPRTES	Hard-to-heal venous leg ulcers	II	Wound bed preparations	[117,118,119]
Pexiganan (MSI-78)	α-helical/+9	Synthetic	GIGKFLKKAKKFGKAFVKILKK	Infected diabetic foot ulcers	III	Topical	[119,120,121]
Melimine	Random coil ^c^/+15	Synthetic	TLISWIKNKRKQRPRVSRRRRRRGGRRRR	Contact lens colonizers, anti-biofilm	II/III	Ocular	[122]
Omiganan (CLS001 or MBI-226)	Linear ^c^/+5	Synthetic	ILRWPWWPWRRK	Skin and catheter infections, antisepsis	III	Topical	[123]
hLF1-11	Linear ^c^/+4	Synthetic	GRRRSVQWCAV	MDR *A. baumannii* MRSA, *Lm*, *E. coli*, and *Kp*	I/II	IV	[124]
Brilacidin (PMX-30063)	Linear/+4	Synthetic	Not available since it is a non-peptide arylamide oligomer	*Staphylococcus* spp. skin infections	II	Topical and mouth rinse	[125]

Abbreviations: hLF1-11: Human lactoferrin 1–11; IV: Intravenous, IP: Intraperitoneal; G-: Gram-negative; G+: Gram-positive; *Kp: Klebsiella pneumoniae*; *Lm: Listeria monocytogenes*; MRSA: Methicillin-resistant *S. aureus*; MDR: multidrug resistant; VRSA: Vancomycin-resistant *S. aureus*. ^a^ Available at http://dramp.cpu-bioinfor.org (accessed on 14 May 2024); ^b^ Available at https://www.clinicaltrials.gov/ (accessed on 14 May 2024); ^c^ May increase their α-helical content/change conformation in bacterial membrane-mimetic environments. The underlined AMPs are FDA-approved.
